# Evaluating Heterodinuclear Mg(II)M(II) (M = Mn, Fe, Ni, Cu, and Zn) Catalysts for the Chemical Recycling of Poly(cyclohexene carbonate)

**DOI:** 10.1021/acscatal.3c04208

**Published:** 2023-11-23

**Authors:** Madeleine L. Smith, Thomas M. McGuire, Antoine Buchard, Charlotte K. Williams

**Affiliations:** † Department of Chemistry, Chemistry Research Laboratory, University of Oxford, 12 Mansfield Rd, Oxford OX1 3TA, U.K.; ‡ Department of Chemistry, University of Bath, Institute for Sustainability, Claverton Down, Bath BA2 7AY, U.K.

**Keywords:** chemical recycling, depolymerization, polycarbonates, polymers, catalysis

## Abstract

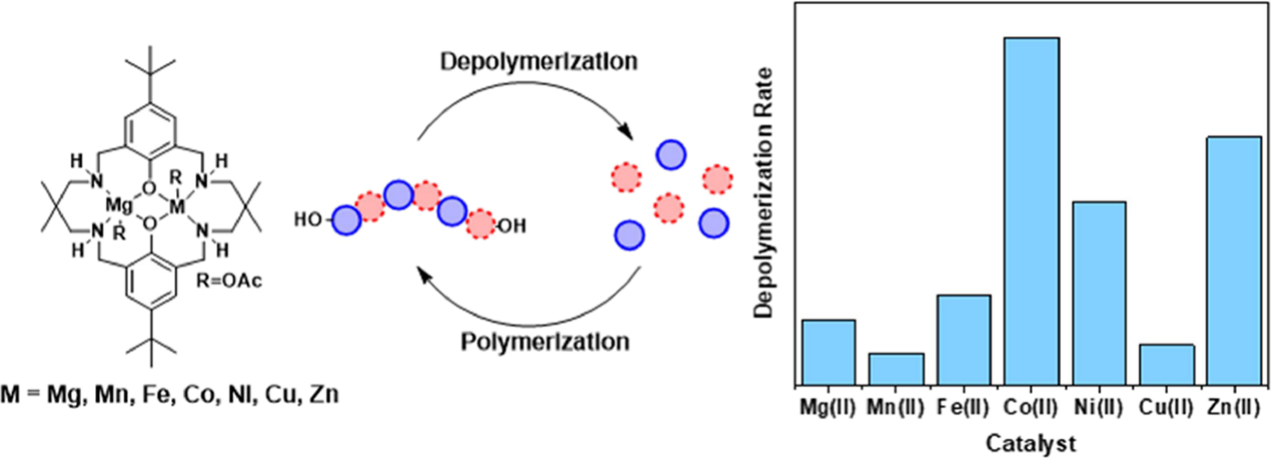

Polymer chemical recycling to monomers (CRM) is important to help achieve a circular plastic economy, but the “rules” governing catalyst design for such processes remain unclear. Here, carbon dioxide-derived polycarbonates undergo CRM to produce epoxides and carbon dioxide. A series of dinuclear catalysts, Mg(II)M(II) where M(II) = Mg, Mn, Fe, Co, Ni, Cu, and Zn, are compared for poly(cyclohexene carbonate) depolymerizations. The recycling is conducted in the solid state, at 140 °C monitored using thermal gravimetric analyses, or performed at larger-scale using laboratory glassware. The most active catalysts are, in order of decreasing rate, Mg(II)Co(II), Mg(II)Ni(II), and Mg(II)Zn(II), with the highest activity reaching 8100 h^–1^ and with >99% selectivity for cyclohexene oxide. Both the activity and selectivity values are the highest yet reported in this field, and the catalysts operate at low loadings and moderate temperatures (from 1:300 to 1:5000, 140 °C). For the best heterodinuclear catalysts, the depolymerization kinetics and activation barriers are determined. The rates in both reverse depolymerization and forward CHO/CO_2_ polymerization catalysis show broadly similar trends, but the processes feature different intermediates; forward polymerization depends upon a metal–carbonate intermediate, while reverse depolymerization depends upon a metal-alkoxide intermediate. These dinuclear catalysts are attractive for the chemical recycling of carbon dioxide-derived plastics and should be prioritized for recycling of other oxygenated polymers and copolymers, including polyesters and polyethers. This work provides insights into the factors controlling depolymerization catalysis and steers future recycling catalyst design toward exploitation of lightweight and abundant s-block metals, such as Mg(II).

## Introduction

Polymer recycling is important to improve resource efficiency, capitalize upon material embedded energy, and reduce greenhouse gas (GHG) emissions associated with polymer manufacturing.^1^ Today’s polymer industry is a significant GHG producer: emitting >1 Gt of CO_2_ equiv. annually and, if sector growth continues unabated, could reach >5 Gt of CO_2_ equiv. by 2050.^2^ Such pollution is unsustainable, and achieving net zero emissions will require major changes to polymer production. Today most polymers are manufactured from fossil chemicals and life cycle assessments reveal that the majority of emissions (60–90%) occur during raw material extraction, chemical processing, and polymer manufacturing, i.e., upstream of use or disposal life cycle stages.^2^ To reduce greenhouse gas emissions, it is essential to both increase plastic waste recycling and to reduce the energy requirements for that recycling.^3^ Depending on the material, a range of recycling technologies are feasible, but mechanical recycling often compromises the polymers’ properties particularly over repeated cycles.^4,5^ Chemical recycling is a process whereby plastics are broken down to their monomers and subsequent repolymerization of these pure monomers should produce materials with equivalent properties to virgin product.^5^ There are a range of different approaches to chemical recycling, but it is perhaps most effectively applied to polymers which can be thermodynamically equilibrated with their monomers, i.e., materials with accessible ceiling temperatures.^6^ Three such classes of polymers are polyesters, -carbonates, and -ethers all of which have promising track records in chemical recycling.^4,5,7−12^ Although the depolymerizations are driven thermodynamically, there are activation barriers that require the use of catalysts.^5^ The current rationale governing catalyst selection for depolymerizations is at a very early stage, lagging behind forward polymerization catalysis. Most reports of CRM focus on individual examples of catalysts without much explanation for how they are “selected” for the target polymer.^5^ Our objective is to develop an understanding of the parameters responsible for an effective depolymerization catalyst and, through use of kinetic measurements, to understand the depolymerization mechanisms.

**Figure 1:**
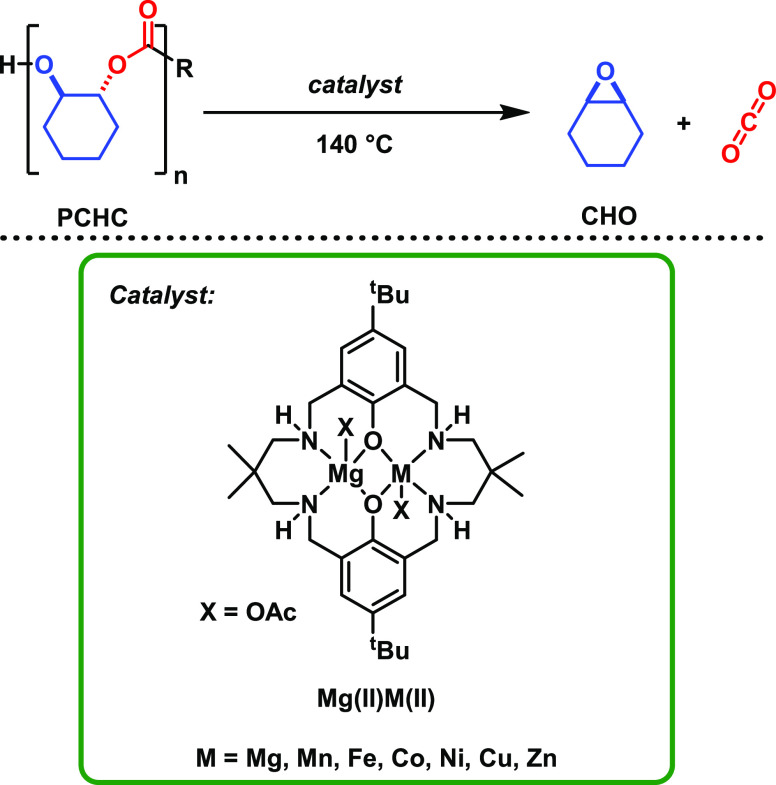
Catalysts for the depolymerization of poly(cyclohexene carbonate) (PCHC) to cyclohexene oxide (CHO) and CO_2_.

This work focuses on the catalyzed CRM of aliphatic polycarbonates prepared from epoxides and carbon dioxide.^13^ The ring-opening copolymerization (ROCOP) of carbon dioxide and cyclohexene oxide produces poly(cyclohexene carbonate) (PCHC); the process depends upon its catalyst and several decades of research have identified some highly active and selective catalysts mostly comprising metal complexes.^14−17^ Epoxide/CO_2_ ROCOP is an interesting method of carbon dioxide utilization and in the case of poly(cyclohexene carbonate), PCHC, up to 31% of the polymer mass derives directly from CO_2_ use.^16,18^ PCHC shows promise as an engineering polymer as it has a high tensile modulus (3.6 GPa) and high glass transition temperature (∼120 °C).^19^ It a useful constituent in block copolymer structures, with the resulting materials’ properties spanning thermoplastic elastomers, thermoplastics and pressure sensitive adhesives depending on the carbon dioxide content.^20−22^ When considering the chemical recycling to monomer of PCHC, an immediate challenge is that the thermodynamic product of the epoxide/CO_2_ coupling is a 5-membered ring cyclic carbonate, cyclohexene carbonate.^15,23^ As such, heating PCHC above its ceiling temperature is likely to form cyclic carbonate rather than the epoxide. *Cis*-cyclohexene carbonate is not a monomer and cannot be repolymerized due to its thermodynamic stability, while *trans*-cyclohexene carbonate requires different polymerization catalysts and conditions to CHO/CO_2_ ROCOP, rendering its incorporation into block copolymers via switch catalysis more challenging. Hence, the development of depolymerization catalysts capable of delivering both high activity and selectivity in the transformation of PCHC into its epoxide (CHO) and carbon dioxide are of high interest (Figure 1).

There are some prior examples for the chemical recycling of polycarbonates to epoxides and carbon dioxide.^24^ In 2013, Darensbourg and co-workers published a ground-breaking report describing solution-state CRM using poly(cyclopentene carbonate) (PCPC).^25^ The catalyst system comprised a [Cr(III)(salen)Cl] complex, applied with ^*n*^Bu_4_NN_3_ as a cocatalyst, which showed excellent selectivity for cyclopentene oxide (∼92%). It was proposed that the relatively high ring-strain of *trans*-cyclopentene carbonate facilitated its depolymerization.^25^ In 2022, Wu and co-workers reported on another PCPC depolymerization catalyst: an organoboron catalyst paired with KOH which produced CPO with >99% selectivity but with a low TOF of 2 h^–1^.^26^ Other research groups have also reported the catalyzed chemical recycling of more specialized polycarbonates to epoxides/CO_2_, including poly(*N*-heterocyclic epoxide carbonates) and poly(limonene carbonate).^27−29^ In 2022, our research team reported the first PCHC CRM using a dinuclear Mg(II)Mg(II) catalyst which selectively produced cyclohexene oxide (CHO) and carbon dioxide.^30^ The process operated in solution (*p*-xylene) and showed good activity, achieving a turnover frequency (TOF) of 150 h^–1^ (1:300 catalyst:PCHC, 120 °C, [PCHC] 1 M in *p*-xylene). The Mg(II)Mg(II) catalyst was also active in forward polymerization, showing high activity and selectivity at low carbon dioxide pressures.^31^ However, other catalysts showing high activity in CHO/CO_2_ ROCOP were completely inactive in chemical recycling.^30^ PCHC depolymerization using a decomposed di-Zn(II) polymerization catalyst was also reported.^32,33^

Solution phase chemical recycling has a number of potential limitations, including the need to isolate the product from the solvent. To address this issue, solid-state chemical recycling processes were investigated. We developed a method to homogeneously disperse the catalyst into the “waste” PCHC by solvent casting “films” for chemical recycling.^34^ These films were evaluated using thermal gravimetric analysis (TGA), under isothermal conditions, and different nitrogen flow rates. Using these methods, a heterodinuclear Mg(II)Co(II) catalyst showed excellent PCHC recycling performance forming CHO in very high selectivity (>99%) and with a very high activity, TOF = 25,700 h^–1^ at 140 °C (cat:PCHC 1:5000, N_2_ flow = 25 mL/min).^34^ In parallel, Lu and co-workers reported the solid-state PCHC thermolysis, catalyzed by a [Cr(III)(salen)Cl]/PPNN_3_ (PPN = bis(triphenylphosphorylidine)ammonium), which showed excellent selectivity (>99%) and a TOF of up to 3000 h^–1^ at 200 °C (cat:PCHC 1:1000).^35^

Here, the chemical recycling of PCHC using a series of dinuclear catalysts in the solid state is investigated. Applying the principle of microscopic reversibility for reactions at equilibrium, we hypothesized that fast forward polymerization catalysts might also show accelerated depolymerization catalysis (i.e., “reverse” reaction). We have previously reported that in forward CHO/CO_2_ ROCOP, some heterodinuclear catalysts outperform homodinuclear analogues, specifically the Co(II)Mg(II) catalyst showed significantly higher ROCOP rates than Mg(II)Mg(II).^5,36−40^

Here, the first objective is to evaluate the depolymerization rates of a series of Mg(II)M(II) catalysts, where M = first row transition metal or Mg(II). A second objective is to understand any trends in the rates and selectivities of the PCHC depolymerizations. The third objective is to compare the performances in “forward” polymerization catalysis with those in “reverse” depolymerization or chemical recycling catalysis.

## Results

A series of heterodinuclear Mg(II)M(II) catalysts, where M = Mn, Fe, Co, Ni, Cu, and Zn, together with the homodinuclear Mg(II)Mg(II) “control” catalyst, were synthesized according to previously described procedures.^40^ All the heterodinuclear complexes were prepared by reacting a macrocyclic ancillary ligand with bis(trimethylsilyl)amido)magnesium(II), followed by the addition of the second metal acetate complex.^40^ The reactions were all selective for the formation of heterodinuclear complexes, which were isolated in high yields and showed characterization data consistent with prior reports.^40^

Dihydroxy-telechelic PCHC was prepared by ROCOP of CHO and CO_2_ (*M*_n,SEC_ = 5400 g mol^–1^, *Đ*_M_ = 1.07, *T*_d,onset_ = 242 °C, and *T*_g_ = 115 °C). Solid-state depolymerization experiments were conducted using isothermal methods with a TGA instrument. The samples for depolymerization were prepared by solvent casting films; a solution of PCHC and the catalyst in THF was added to a TGA pan and dried in vacuo for 30 min to remove all solvent. Isothermal depolymerizations were conducted at 140 °C, using a nitrogen flow of 25 mL/min, with monitoring of the PCHC mass loss over time. The reaction temperature (140 °C) was selected as the optimum as it is sufficiently above the PCHC glass transition temperature (*T*_g_ = 115 °C) to ensure effective mass transport and should reach high rates with minimized energy demand. A catalyst loading of [cat]:[PCHC]_0_ 1:300, where [PCHC]_0_ refers to the concentration of polymer repeat units, was used to ensure comparisons could be made between the heterodinuclear Mg(II)M(II) catalysts and Mg(II)Mg(II) catalyst previously reported.^30,34^ Control experiments to determine catalyst stability showed all the complexes were stable at 140 °C for >2 h (Figure S4). Another control experiment revealed no mass loss when PCHC was heated at 140 °C for >2 h without any catalyst (Figure S5).

**Figure 2:**
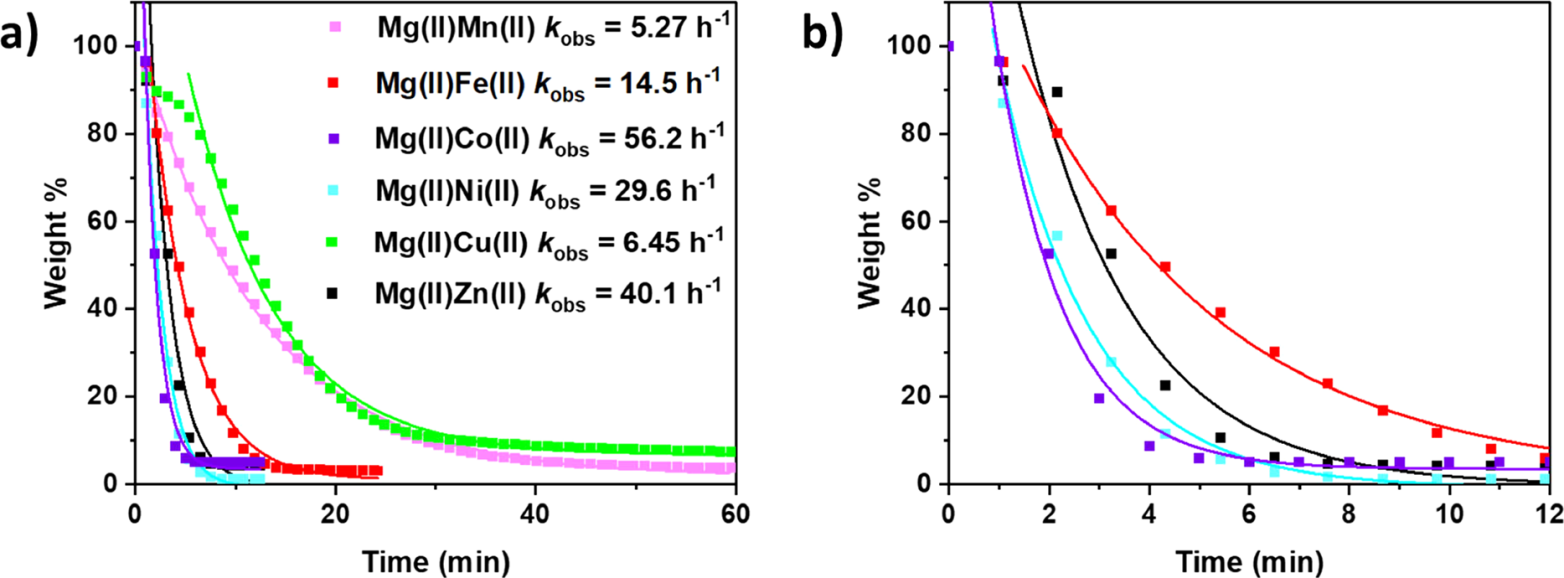
(a) Weight % vs time using different Mg(II)M(II) catalysts, with data fitted to exponential decays. Depolymerizations were conducted under a N_2_ flow rate of 25 mL min^–1^, at 140 °C, [Cat]_0_: [PCHC]_0_ 1:300 (see SI for the detailed experimental setup). (b) Expanded region of the data set shown in (a).

**Figure 3:**
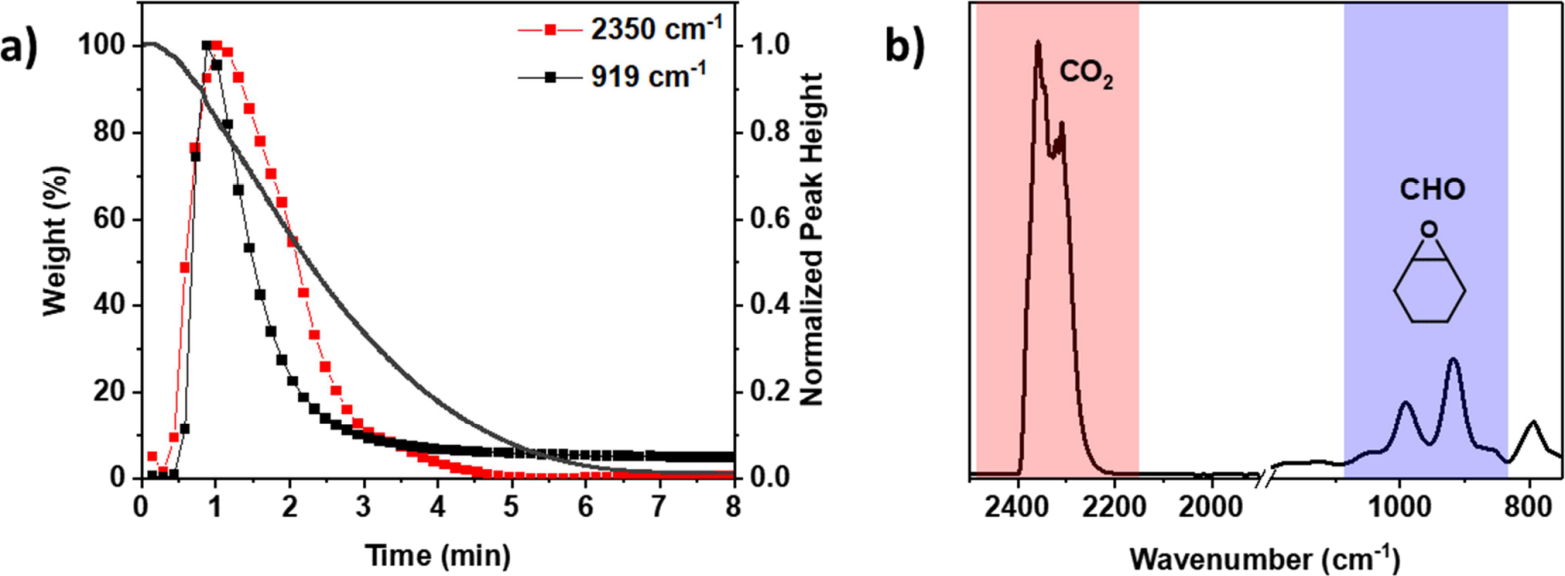
(a) Weight % (left axis) and normalized peak height (right axis, black line = 919 cm^–1^, red = 2350 cm^–1^) vs time data for the depolymerization of PCHC. Reaction conditions: PCHC:Mg(II)Co(II) 300:1, 140 °C, N_2_, and monitored by IR spectroscopy. The peaks with wavenumbers of 919 and 2350 cm^–1^ are assigned as CHO and CO_2_, respectively. (b) IR spectrum for the depolymerization of PCHC with regions corresponding to CO_2_ and CHO highlighted.

All catalysts successfully depolymerized PCHC, reaching >90% mass loss in <20 min and the fastest catalysts achieved >90% mass loss in <5 min (Table 1). All of the catalysts displayed exponential decreases in mass over time, consistent with first-order rate dependencies (Figure 2). The pseudo-first-order depolymerization rates, *k*_obs_, were determined from exponential fits to the mass loss data and, in each case, showed *R*^2^ > 0.98 (Figure 3). All reactions were repeated in triplicate to allow for determination of error ranges. The *k*_obs_ and point-kinetic (TOF) data allowed for comparisons between the different catalysts (Table 1). The Mg(II)Co(II), Mg(II)Ni(II) and Mg(II)Zn(II) catalysts showed the highest activities with TOF values of 8100 ± 780, 5500 ± 300 and 5600 ± 800 h^–1^, respectively. These are fully consistent with the depolymerization rate constant values of 56.2 ± 5, 29.6 ± 0.9, and 40.1 ± 6 h^–1^. The Mg(II)Fe(II) catalyst was slower, with a TOF of 2000 ± 80 h^–1^, and the two slowest catalysts were Mg(II)Mn(II) and Mg(II)Cu(II) which showed much lower TOF values of 620 ± 140 and 830 ± 60 h^–1^, respectively (Table 1). These slowest catalysts showed significant polymer residual mass which may indicate some polyether formation. Overall, the “second” transition metal appears to exert a significant influence over the depolymerization rates with the order being Co(II) > Zn(II)=Ni(II) > Fe(II) > Mn(II) > Cu(II).

**Table 1:** Data for the Depolymerizations Using Mg(II)M(II) Catalyst and Previously Reported Catalysts for the Solid-State Reaction.^a^

entry	Mg(II)M(II)	time (s)^b^	PCHC conversion %^c^	TOF (h^–1^)^d^	*k*_obs_ (h^–1^)^e^	mass loss rate (kg g^–1^ h^–1^)^f^
1	Mg(II)Mg(II)	416 ± 23	97 ± 0.1	1600 ± 90	10.5 ± 0.8	0.310 ± 0.02
2	Mg(II)Mn(II)	1110 ± 270	95 ± 3	620 ± 140	5.27 ± 0.6	0.120 ± 0.03
3	Mg(II)Fe(II)	330 ± 13	99 ± 0.1	2000 ± 80	14.5 ± 0.2	0.370 ± 0.02
4	Mg(II)Co(II)	81 ± 7	98 ± 0.5	8100 ± 780	56.2 ± 5	1.50 ± 0.1
5	Mg(II)Ni(II)	119 ± 6	99 ± 0.5	5500 ± 300	29.6 ± 0.9	1.03 ± 0.05
6	Mg(II)Cu(II)	783 ± 52	96 ± 0.3	830 ± 60	6.45 ± 0.4	0.160 ± 0.01
7	Mg(II)Zn(II)	119 ± 20	98 ± 0.4	5600 ± 800	40.1 ± 6	1.05 ± 0.2

a Depolymerizations conducted using TGA measurements, under a N_2_ flow rate of 25 mL min^–1^, at 140 °C, [Cat]_0_: [PCHC]_0_ 1:300 (see SI for the detailed experimental setup).

b Time from 20 to 80% mass loss. Average error taken from repeated runs.

c PCHC conversion corresponds to the total weight loss of PCHC at the end of the depolymerization reaction.

d TOF = (moles of PCHC consumed (20–80% conversion)/mol of catalyst/time.

e *k*_obs_ calculated from first-order exponential decay fits of mass % vs time plots.

f Mass loss rate = (mass of PCHC consumed (20–80% conversion)/mass of catalyst/time.

To better understand the performances of the heterodinuclear catalysts, they were compared to the homodinuclear Mg(II)Mg(II) catalyst. The Mg(II)Mg(II) catalyst showed a TOF of 1600 ± 90 h^–1^ and *k*_obs_ = 10.5 ± 0.8 h^–1^ which places it between Mn(II) and Fe(II) in the series above, i.e., catalysts featuring Fe(II), Zn(II), Ni(II), and Co(II) are all more active than Mg(II)Mg(II), but those featuring Cu(II) and Mn(II) are less active. The most active catalyst, Mg(II)Co(II), is ∼5× more active than Mg(II)Mg(II). Therefore, considering all of the catalysts tested, the order of depolymerization rates follows the trend:
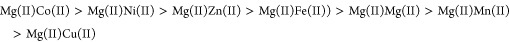


To confirm the selectivity for cyclohexene oxide formation, TGA-IR, TGA-MS and product isolation experiments were performed (see the SI for experimental details). The TGA-IR data showed peaks at 2350 and 919 cm^–1^, assigned to carbon dioxide and CHO, respectively (Figure 3). All catalysts displayed high selectivity forming >99% CHO and ∼0.3% *trans* CHC, as determined by ^1^H NMR spectroscopy of the isolated product (Figure S6). Moreover, regular product isolation revealed that the CHO selectivity remained both very high and constant as the depolymerization progressed (Figure S8).

To understand any practical uptake of the catalysis, the activity can also be defined in terms of the mass loss rate (Table 1). The Mg(II)Co(II) species is the best performing catalyst, displaying a mass loss rate of 1.50 ± 0.1 kg PCHC/g catalyst. An earlier investigation demonstrated the potential to “recycle” the Mg(II)Co(II) catalyst by repeatedly adding fresh “batches” of PCHC to catalyst residues, without compromise to either productivity or selectivity.^19,34^ Thus, Mg(II)Co(II) is recommended for scale-up testing, followed by Mg(II)Zn(II) and Mg(II)Ni(II).

**Figure 4:**
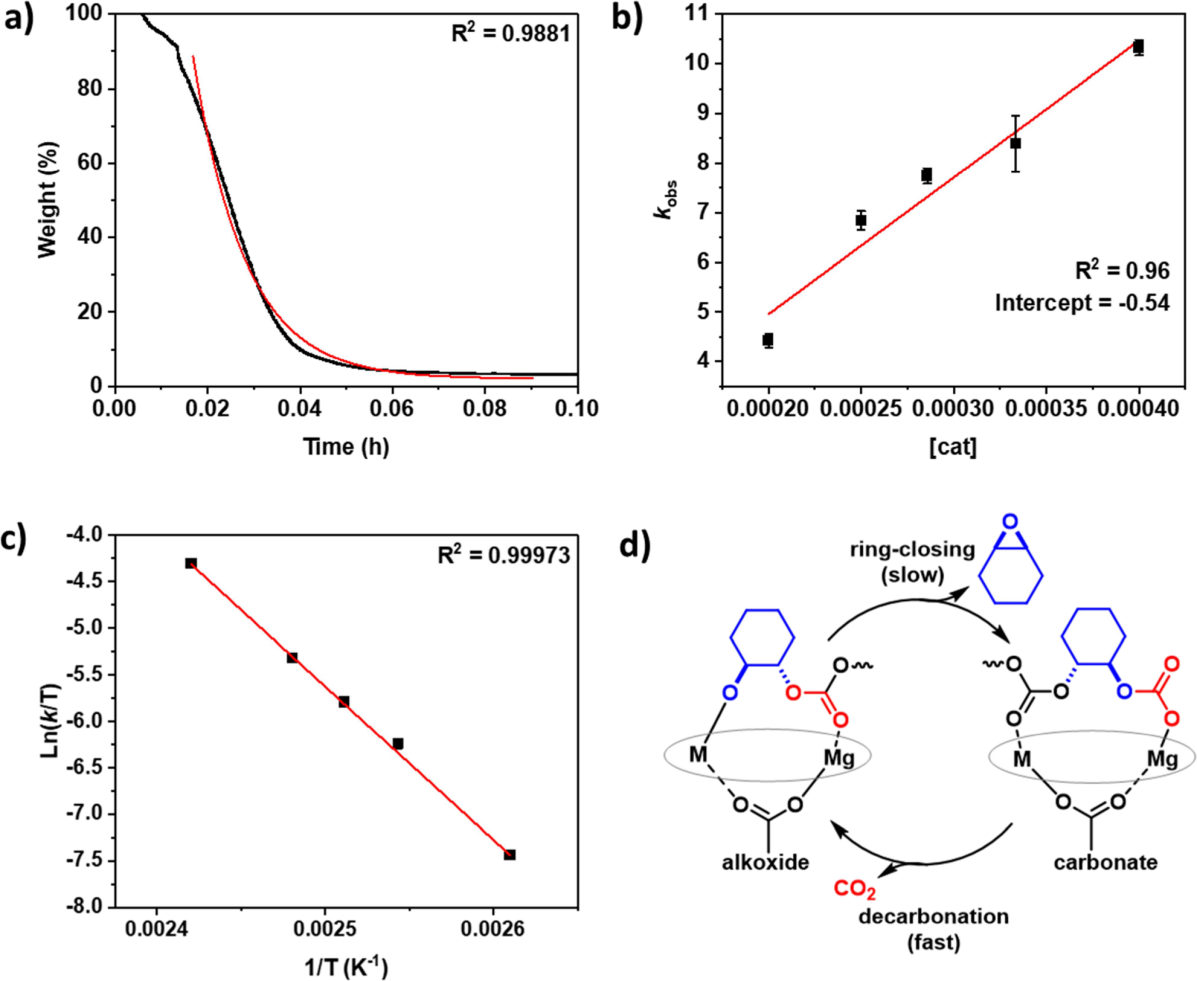
Data used to determine the depolymerization rate law for PCHC using the Mg(II)Co(II) catalyst. (a) Weight % vs time for Mg(II)Co(II) catalyst, with data fit to an exponential decay. Depolymerizations conducted under a N_2_ flow rate of 25 mL min^–1^, at 140 °C, [Cat]_0_: [PCHC]_0_ 1:300 (see SI for the detailed experimental setup). (b) Plot of *k*_obs_ vs [cat]. Depolymerizations performed under a N_2_ flow rate of 25 mL min^–1^ at 140 °C. (c) Eyring analysis conducted under a N_2_ flow rate of 25 mL min^–1^ between 100–140 °C, [Cat]_0_:[PCHC]_0_ 1:2500. *k*_obs_ values calculated from the gradient of the ln(weight/weight_0_) vs time plot at 50% PCHC weight loss. (d) Proposed depolymerization mechanism.

Next, the depolymerization kinetics were evaluated for the Mg(II)Co(II) catalyst. All catalysts showed exponential mass loss vs time data, consistent with rates having a first order dependence on PCHC mass (Figure 4a). To determine the order in catalyst concentration, depolymerizations were conducted using [Mg(II)Co(II)]_0_:[PCHC]_0_ of 1:2500, 1:3000, 1:3500, 1:4000, and 1:5000, with *k*_obs_ determined from exponential fits. The plot of the rate constant, *k*_obs_, vs catalyst concentration showed a linear fit (Figure 4b). The linear fit is consistent with the depolymerization rate being first order in the catalyst concentration. Other fits, corresponding to a half order or second order in catalyst concentration, were shown to be inferior (Figures S15–S17). The gradient of this plot allows for the determination of *k*_d_, the depolymerization rate constant. Overall, PCHC depolymerization follows a second-order rate law, which is first order in both catalyst and polymer mass:
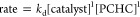


The rate law is assumed to apply to all other catalysts in the series, given that the catalyst structure differs only in the selection of the second metal. There is no evidence of aggregation by CV, IR or MALDI-TOF. Furthermore, all catalysts show exponential decays in the mass loss vs time plots, suggesting a common rate law. This rate law is interpreted by a chain backbiting mechanism (Figure 4d). As such, the polycarbonate hydroxyl end-groups react with the catalyst to form a Mg(II)Co(II) alkoxide intermediate. This alkoxide species is the “true catalyst” and undergoes chain backbiting to form the epoxide (CHO) and the Mg(II)Co(II) carbonate intermediate. The carbonate intermediate undergoes decarboxylation, releasing carbon dioxide, to reform a “chain-shortened” Mg(II)Co(II) alkoxide intermediate. From this point, the alkoxide intermediate reacts with the chain again, and the depolymerization cycle continues, until the whole chain is consumed. It is proposed that the metal alkoxide “backbiting” or nucleophilic attack is rate limiting. The proposed mechanism is consistent with the rate law since the rate determining step should depend upon both the catalyst and polymer mass. Earlier work showed that no depolymerization occurred with acetyl-end-capped PCHC (as opposed to hydroxyl-end-capped PCHC). Given that activity is only observed when metal-alkoxide formation is possible, i.e. with hydroxyl-end-capped PCHC, these results support our hypothesis that the metal-alkoxide intermediate is the true catalyst and therefore essential in rationalizing catalytic performance in depolymerization.^34^ It is tentatively proposed that one of the acetate coligands remains coordinated to the complex throughout the depolymerization reaction. Analysis of the Mg(II)Co(II) catalyst after the depolymerization by MALDI-ToF and IR confirmed the presence of an acetate ligand (Figures S10 and S11).^34^

Given the significant impact of the second transition metal on the depolymerization rates in the series of heterodinuclear catalysts, it is tentatively proposed that the alkoxide might be primarily coordinated to M(II) (rather than Mg(II)). Eyring analysis was performed using the Mg(II)Co(II) (Figure 4c). Accordingly, depolymerization rates were determined from a series of experiments conducted at temperatures from 110 to 140 °C, at a fixed catalyst:PCHC of 1:2500. Plots of ln(*k*_d_/*T*) vs 1/*T* allowed for determination of Δ*H*^‡^ (gradient) and Δ*S*^‡^ (*y*-axis intercept) and for Δ*G*^‡^ (Figure 4c). The catalyst showed a positive transition state entropy (Δ*S*^‡^ = 97.6 ± 1.7 J mol^–1^) that could indicate a dissociative mechanism. The positive transition state entropy also indicates a preordered transition state consistent with the rate determining step involving the release of a molecule of epoxide, together with the polymer-complex formation.

## Discussion

The series of dinuclear catalysts shows significant potential in the depolymerization of PCHC to form CHO and carbon dioxide. They are applied in the solid state, by forming polymer/catalyst films, at 140 °C and show near complete selectivity for CHO formation. The catalysts showed different rates, with the most active species comprising Co(II), Ni(II) and Zn(II) combined with Mg(II). The catalysts were also successfully applied at loadings ranging from 1:300 to 1:5000. The depolymerization rate law is first order in polymer mass and first order in catalyst concentration. The process is proposed to occur via a rate determining step in which the M(II)-alkoxide intermediate attacks the chain-end extruding a molecule of epoxide and forming a metal–carbonate intermediate.

These dinuclear catalysts showed better performance than other reported PCHC depolymerization catalysts. They are effective at considerably lower temperatures than the [Cr(III)(salen)Cl]/PPNN_3_ catalyst system and at lower loadings (200 °C, catalyst:PCHC 1:1000, TOF = 3000 h^–1^).^35^ Under comparable conditions at 140 °C, the Cr(III) catalyst system reached only 5% PCHC conversion. Liao and co-workers reported a Zn(II) catalyst, formed by the partial hydrolysis of (BDI-ZnMe_2_) (BDI = β-diketiminato), which showed a TOF of 2.5 h^–1^, at 150 °C (catalyst:PCHC 1:50).^32,33^ Overall, compared to the other known polycarbonate chemical recycling catalysts, these dinuclear catalysts benefit from being single component enabling them to be applied at lower loadings, operate at low temperatures, and show field-leading activity values.

**Figure 5:**
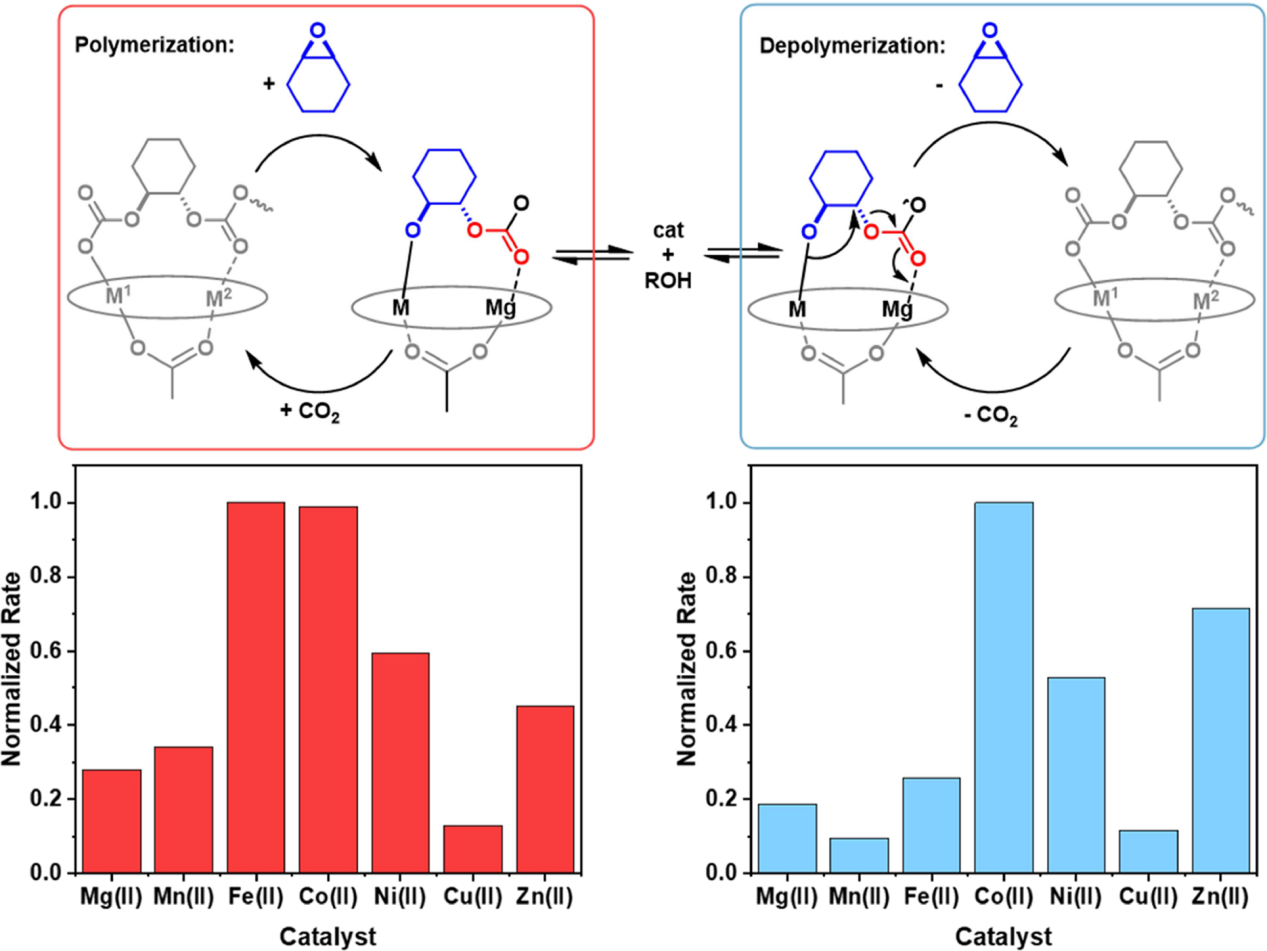
Normalized *k*_obs_ values for the series of Mg(II)M(II) catalysts for the depolymerization of PCHC (140 °C, [cat]_0_:[PCHC]_0_ = 1:300) and the polymerization of CHO/CO_2_ (1 bar CO_2_, 100 °C, [cat]_0_:[CHD]_0_:[CHO]_0_ = 1:20:4000) obtained from previous work.^40^

The final objective of the investigation was to understand whether there were any correlations between “high-performance” forward polymerization (CHO/CO_2_ ROCOP) and depolymerization (PCHC recycling) catalysts. Due to the different reaction conditions of the polymerization and depolymerization, rates were normalized relative to the fastest catalyst for each reaction to allow for comparison. The polymerization reaction was performed under a CO_2_ atmosphere at 100 °C, [cat]:[CHO]_0_ 1:4000, while the depolymerization reaction was performed under N_2_ at a higher temperature of 140 °C, [cat]:[PCHC]_0_ 1:300. This series of heterodinuclear Mg(II)M(II) catalysts shows broadly similar trends for the polymerization and depolymerization reactions (Figure 5). In both reactions, the Mg(II)Co(II), Mg(II)Ni(II), Mg(II)Fe(II), and Mg(II)Zn(II) catalysts are markedly faster than the Mg(II)Mn(II) and Mg(II)Cu(II) catalysts.^40^

These “top 4” complexes all showed higher activities than the Mg(II)Mg(II) catalyst in both polymerization and depolymerization. In both forward and reverse directions, the fastest catalyst is the Mg(II)Co(II) complex. The Mg(II)Fe(II) catalyst warrants some further discussion since it performs “second best” in forward polymerization but is much less effective than the Mg(II)Co(II) complex in depolymerization catalysis. Analysis of postcatalysis samples suggests it undergoes some decomposition after catalysis (Figures S12–14).

The epoxide/carbon dioxide ROCOP and PCHC depolymerizations have different rate determining intermediates in the mechanisms. In the forward reaction, as established over the past decade,^37,41,42^ the metal–carbonate species is rate limiting, while in the reverse reaction, the metal-alkoxide intermediate is rate limiting. This difference in chemistry could rationalize differences between forward and reverse reactions since the “trends” governing the reactivity of such intermediates, as well as their relative energies, are not expected to be completely identical.

**Figure 6:**
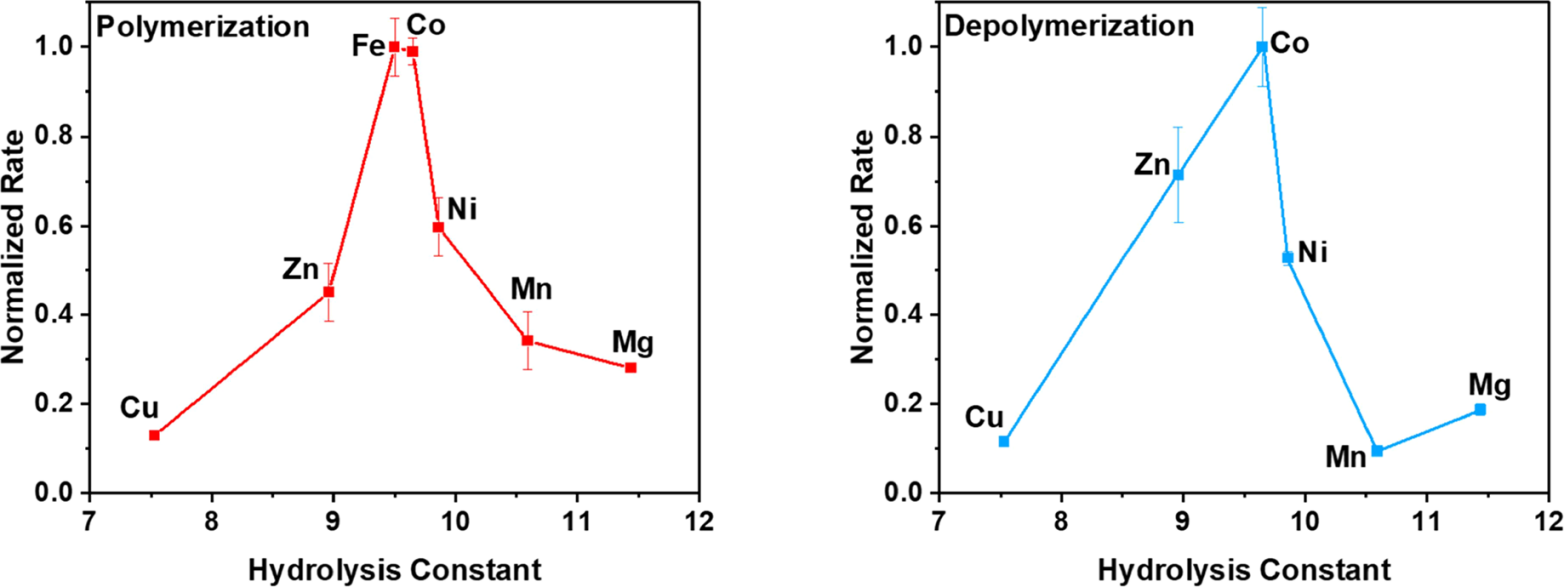
Relationship between the metal hydrolysis constant and the normalized *k*_obs_ values for the series of Mg(II)M(II) catalysts for the depolymerization of PCHC (140 °C, [cat]_0_:[PCHC]_0_ = 1:300) and the polymerization of CHO/CO_2_ (1 bar CO_2_, 100 °C, [cat]_0_:[CHD]_0_:[CHO]_0_ = 1:20:4000) obtained from previous work.^40^ The Mg(II)Fe(II) catalyst has been removed from the plot for depolymerization as the catalyst was shown to be unstable. Hydrolysis constants obtained from ref (43).

In polymerization, it is proposed that the transition metal–carbonate attacks a Mg(II) coordinated CHO molecule,^37^ while in depolymerization, it is tentatively proposed that the Mg(II) coordinates to the carbonyl of a carbonate group adjacent to the chain end and the transition metal-alkoxide attacks and extrudes a CHO molecule. Thus, in both processes, the transition metal–oxygenate nucleophilicity appears to be important in governing activity. To probe this further, various proxies for metal–oxygenate nucleophilicity (including transition metal Lewis acidity, oxophilicity, electronegativity, bond dissociation energy, ionic radius, hydrolysis constant, and water-exchange rate constant) were plotted against the normalized rate for both depolymerization and polymerization. The plot of normalized rate against hydrolysis constant, which acts as a good proxy for metal Lewis acidity, displays a volcano relationship for both depolymerization and polymerization (Figure 6 and Table S1).^43^ No clear correlations could be identified for the other parameters investigated, but as the rate-determining step depends on multiple factors, this is perhaps to be expected (Figure S19).

The optimum transition metal has an intermediate hydrolysis constant. This suggests that to maximize rate in both polymerization and depolymerization, there must be a compromise between the nucleophilicity of the metal–oxygenate and the Lewis acidity of the metal. As the rate-determining step for both polymerization and depolymerization involves nucleophilic attack by a metal–oxygenate species, a more nucleophilic metal–oxygenate should give a faster rate. At higher hydrolysis constants, the metals are weaker Lewis acids and the metal–oxygenate species are relatively destabilized, resulting in a more nucleophilic, and therefore more reactive, metal–oxygenate. However, the Lewis acidity of the metal is also important. A more Lewis acidic metal promotes the insertion of the catalyst into the OH groups of the terminal hydroxyl groups of the polymer, driving the formation of the reactive metal-alkoxide species. By increasing the concentration of the metal-alkoxide species, the equilibrium between the metal-alkoxide and metal–carbonate species is driven toward formation of the metal–carbonate. This is important in both depolymerization and polymerization reactions. In depolymerization the increased metal-alkoxide concentration drives the backbiting reaction to form a metal–carbonate, while in polymerization the formation of the reactive metal–carbonate is promoted. Therefore, a balance is needed between the Lewis acidity of the metal and the nucleophilicity of the metal–oxygenate. It should be noted that in the depolymerization the Mg(II)Fe(II) catalyst does not fit the observed trend due to catalyst decomposition.

Eyring analysis reveals that for depolymerization, in contrast to the polymerization, the transition state entropy values are positive. This suggests that the depolymerization proceeds via a dissociative mechanism and reflects how depolymerization is entropically driven.

## Conclusions

A series of dinuclear Mg(II)M(II) catalysts, where M(II) = Mg, Mn, Fe, Co, Ni, Cu, and Zn, were investigated for the solid-state depolymerization of poly(cyclohexene carbonate) to cyclohexene oxide and CO_2_. The catalysts formed epoxide with >99% selectivity and showed some of the highest activity values in the field, with TOF up to 8100 h^–1^. The best catalysts were Mg(II)Co(II), Mg(II)Ni(II), and Mg(II)Zn(II). Depolymerization kinetics supported a chain backbiting mechanism in which rates are controlled by a metal-alkoxide attacking its neighboring group in the polymer chain to extrude the cyclohexene oxide.

Comparing the catalysts for both forward polymerization and reverse “depolymerization” revealed broadly similar performances, but the key intermediates in forward and reverse reactions are different. This difference is a direct consequence of these polymers being prepared from 2 monomers (copolymerization). More generally, these findings suggest that effective forward polymerization catalysts should be prioritized for applications in “reverse” recycling processes. In polycarbonate chemical recycling, these dinuclear catalysts showed field-leading rates and selectivity. For recycling, they are highly active and selective and operate at low temperatures. In the future, these catalysts should be investigated for the chemical recycling of other polycarbonates, including biobased poly(limonene carbonate) and block polycarbonates. The catalysts showed highly reactive “alkoxide” intermediates and so should also be prioritized for the chemical recycling of polyesters and polyethers.

## Supporting Information Available

Experimental methods, characterization, and polymer/monomer purity evaluation data (PDF)
