# Supplementary material for: Evaluating Heterodinuclear Mg(II)M(II) (M = Mn, Fe, Ni, Cu, and Zn) Catalysts for the Chemical Recycling of Poly(cyclohexene carbonate)
Source: ACS Catal. 2023 Nov 23;13(24):15770–8. doi: 10.1021/acscatal.3c04208 (PMC10728899; doi:10.1021/acscatal.3c04208)
Supplement: Supplementary file 1 — cs3c04208_si_001.pdf [file cs3c04208_si_001.pdf]

Supporting Information:

Evaluating Heterodinuclear Mg(II)M(II) (M = Mn, Fe, Ni, Cu and Zn) Catalysts for the Chemical Recycling of Poly(Cyclohexene Carbonate)

Madeleine L. Smith[a], Thomas M. McGuire,[a] Antoine Buchard[b] and Charlotte K. Williams[a*]

[a]Department of Chemistry, Chemistry Research Laboratory, University of Oxford, 12 Mansfield Rd, Oxford, OX1 3TA, U.K

[b]Department of Chemistry, University of Bath, Institute for Sustainability, Claverton Down, Bath BA2 7AY, U.K.

Corresponding Authors

Charlotte K. Williams: Email: charlotte.williams@chem.ox.ac.uk

Contents

1. Materials and Methods 5

1.1 Catalyst Synthesis 5

Scheme S1. Mg(II)M(II) Catalyst Synthesis. 5

1.2 Synthesis of Poly(cyclohexene carbonate) (PCHC) 5

1.3 Solid-State PCHC Depolymerization 6

Scheme S2. Solid-state PCHC depolymerization set up for TGA. 6

1.4 Product Isolation after Depolymerization 6

Figure S1. Product isolation after depolymerization by collecting products from the TGA exhaust in a cold trap. 7

1.5 Monitoring of Depolymerization 7

1.6 Catalyst Stability Tests 7

1.7 PCHC Stability Test 7

1.8 Eyring Analysis for TGA Monitored Depolymerization 7

1.9 Characterization of Mg(II)Fe(II) Catalyst Post Heating and Depolymerization 9

2. PCHC Characterization 9

Figure S2. SEC data for the PCHC 9

Figure S3. ^{1}H NMR Spectrum ($CDCl_3$) for the PCHC used in all depolymerizations. 9

3. Depolymerization Catalysis Data 10

Figure S4. Mass loss vs time plots for catalysts demonstrating their stability. 11

Figure S5. Mass loss vs time plots for PCHC with no catalyst present 12

Figure S6. ^{1}H NMR Spectrum ($CDCl_3$) of CHO, isolated from the depolymerization of PCHC. 12

Figure S7. Mass loss and normalized ion intensity vs time for the depolymerization of PCHC 13

Figure S8. Identification of product distribution by isolation against % weight of PCHC 14

Figure S9. Mass loss vs time data for the depolymerization of PCHC fitted to a first order exponential decay 16

Figure S10. MALDI-TOF spectrum of catalyst before and after depolymerization 17

Figure S11. IR spectra of Mg(II)Co(II) before and after depolymerization 17

Figure S12. IR Spectra of the Mg(II)Fe(II) catalyst characterized after heating and post depolymerization reaction 18

Figure S13. Cyclic voltammograms for the Mg(II)Fe(II) catalyst after heating at 140 °C and post depolymerization reaction. 19

Figure S14. MALDI-ToF spectra for the Mg(II)Fe(II) catalyst after heating and post depolymerization reaction 20

Figure S15. Plot of k_{obs} vs. [cat] with fittings 21

Figure S16. Determination of the order in catalyst concentration. Plot of lnk_{obs} vs ln[cat] 22

Figure S17. Determination of the order in catalyst concentration. Plot of lnk_{obs} vs ln[cat] with fittings....................22

Figure S18. Depolymerizations of PCHC using the Mg(II)Co(II) catalyst and data used for Eyring analysis 24

Table S1. Literature values for the hydrolysis constants of the M(II) cations. 25

Figure S19. Plots of the normalised k_{obs} for depolymerization against various proxies for metal alkoxide nucleophilicity 25

4. References 26

1. Materials and Methods

All experiments were carried out under N_2 using standard Schlenk/glovebox techniques unless otherwise stated. Cyclohexene oxide was purchased from commercial sources (Acros organics) and used as received. All solvents used were anhydrous, unless otherwise stated. THF and toluene were obtained from an SPS system, degassed by several freeze-pump-thaw cycles and stored over 3 Å molecular sieves, under nitrogen. 1,2-*trans*-Cyclohexenediol (Sigma Aldrich) was recrystallised from anhydrous ethyl acetate. Research-grade carbon dioxide was dried through a Drierite column and two additional drying columns (Micro Torr, Model number: MC1-804FV) in series before use. The catalyst, L^ICo(III)K(I), used to prepare poly(cyclohexene carbonate) (PCHC) was synthesised and used according to literature procedures.[1]

Size exclusion chromatography (SEC) was carried out on a Shimadzu LC-20AD instrument using two PSS SDV linear M columns in series, with a THF eluent. Measurements were conducted at 30 °C, with a flow rate of 1 mL/min. Samples were detected with a differential refractive index (RI) detector. Number-average molar mass ($M_{n,SEC}$),and dispersities, ($Đ_M = M_w/M_n$)) were calculated against a polystyrene calibration. The polymer samples were dissolved in HPLC-grade THF, at a concentration of *ca* 10 mg/mL, and filtered through a 0.2 μm microfilter prior to analysis

Thermal gravimetric analysis (TGA) was performed on a TGA/DSC 1 system (Mettler-Toledo Ltd). Details of depolymerization experiments conducted on the TGA are given in the methods section.

NMR spectra were obtained using a Bruker AVIII HD nanobay NMR spectrometer. Coupling constants are given in Hertz. Selectivities were determined by ^{1}H NMR spectroscopy.

Turnover Frequency (TOF) calculations were performed using mass loss against time plots from 20-80% mass loss of the polymer over time. To account for any residual solvent loss prior to depolymerization (approx. 10 % of mass), the polymer + catalyst sample was compared against a control sample featuring only the polymer.[2]

1.1 Catalyst Synthesis

The macrocyclic diphenol tetramine-based ligand (H_2L) was prepared according to the literature procedure.[3]

The catalyst synthesis followed the procedure previously published reported (Scheme S1).[4] Under inert conditions, [Mg(N(Si(CH$_3$)$_3$)$_2$)$_2$.THF] (0.44 g, 0.91 mmol) was added to H_2L (0.5 g, 0.91 mmol), in THF (15 mL), and stirred for 2 h. M(OAc)$_2$ (0.91 mmol) was added to the reaction solution and stirred for 16 h, at 100 °C, in a J-Young ampoule. The solution was reduced to dryness *in vacuo* and the product was washed with hexane (3 x 20 mL) to afford the final complex. All catalysts were characterized by IR spectroscopy, MALDI-ToF, cyclic voltammetry and elemental analysis. Characterization data was consistent with prior reports.[4]

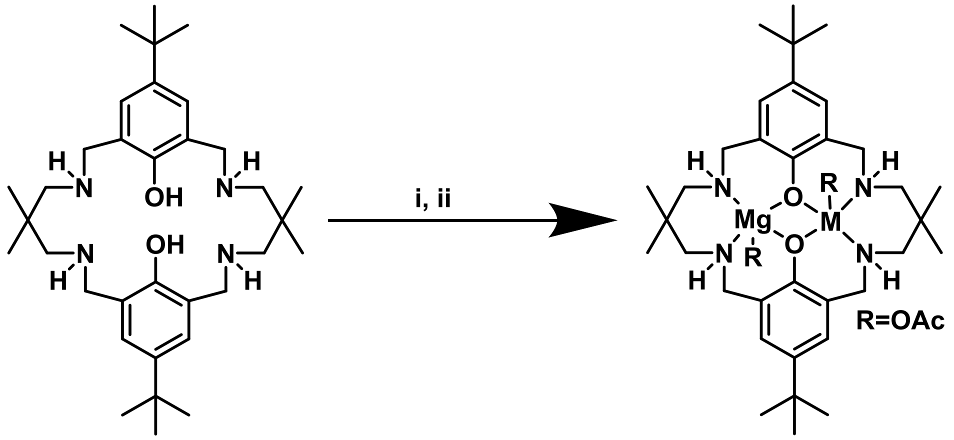

Scheme S1. Mg(II)M(II) Catalyst Synthesis.

i) Mg[N(SiMe$_3$)$_2$]$_2$, THF, 25 °C, N_2, 2 h ii) M(OAc)$_2$, THF, 100 °C, N_2, 16 h.

1.2 Synthesis of Poly(cyclohexene carbonate) (PCHC)

Cyclohexene oxide (50 mL, 494 mmol, 10,000 equiv.), 1,2- *trans*-cyclohexanediol (0.57 g, 4.94 mmol, 100 equiv.) and $L^ICo(III)K(I)(OAc)_2$ catalyst (0.03 g, 0.05 mmol, 1 equiv.) were loaded into the 100 mL reactor. The reaction mixture was heated to 50°C under a 30 bar pressure of CO_2. The polymerization was quenched with acetic acid (2 mL) and the polymer precipitated from methanol (250 mL). The precipitation was repeated 3 times to remove the catalyst and isolate the polymer. The polymer was dried, under vacuum, to yield PCHC as a white powder (23.3 g, 33.2 % yield).

$\boldsymbol{M}_{\mathbf{n},\text{SEC}}$ = 5400 g mol^{-1} (1.07)

$\boldsymbol{T}_{\mathbf{d},\text{onset}}$ = 242 °C

$\boldsymbol{T}_{\mathbf{g}}$ = 115 °C

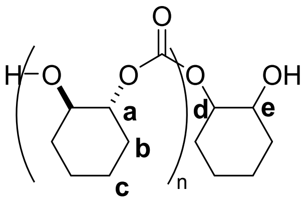

^{1}H NMR (400 MHz, $CDCl_3$) δ 4.82 – 4.51 (m, 2H, **H_a**), 4.49 – 4.32 (m, end group, **H_d**), 3.70 – 3.48 (m, end group, **H_e**), 2.25 – 1.93 (m, 2H, **H_b**), 1.85 – 1.61 (m, 2H, **H_c**), 1.60 – 0.98 (m, 4H, **H_b, H_c**).

1.3 Solid-State PCHC Depolymerization

In the glovebox, PCHC (142 mg, 1.00 mmol), dissolved in THF (1 mL), was added to a vial containing the Mg(II)M(II) catalyst (0.3 mmol). The catalyst:polymer stock-solution (40 µL) was transferred to an aluminium TGA crucible. The crucible was placed under vacuum, for 30 minutes, before being crimped in the glovebox with a hermetic seal. The crucible was then transferred to a TGA instrument for solid-state depolymerization using the method outlined below.

1. N_2 flow of 25.0 mL min^{-1}
2. Equilibrate at 30 °C
3. Heat to 140 °C
4. Isotherm at 140 °C, whilst monitoring mass loss
5. After 1 h (> 95 % mass loss in all cases), sample was cooled to 30 °C

At the start of each TGA run, the crucible was pierced and immediately placed under a flow of N_2. The piercing of the crucible allows for the volatile reaction products (in this case CHO and CO_2) to be released from the pan under the flow of N_2, helping to favour the equilibrium towards depolymerization. The use of TGA enables PCHC mass loss to be monitored over time.

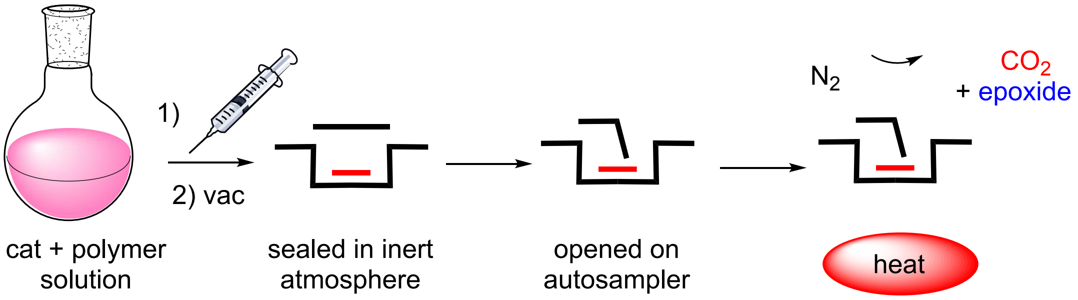

Scheme S2. Solid-state PCHC depolymerization set up for TGA.

1.4 Product Isolation after Depolymerization

A round-bottomed flask was attached to the outlet of the TGA instrument. The 2-necked round-bottomed flask was cooled, over liquid nitrogen, to trap any products. ^{1}H NMR spectroscopy was used to identify the product selectivity.

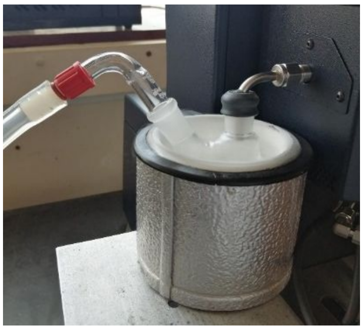

Figure S1. Product isolation after depolymerization by collecting products from the TGA exhaust in a cold trap.

1.5 Monitoring of Depolymerization

In the glovebox, PCHC (426 mg, 3.00 mmol) and Mg(II)Zn(II) (7.5 mg, 0.01 mmol) were added to a pestle and mortar. The mixture was ground to a fine powder and transferred to platinum crucibles. The crucibles were transferred to the TGA instrument. The depolymerization reaction was stopped at fixed % mass loss values of 10, 20, 30, 40, 50, 60 ,70, 80 or 90 % and the remaining pan contents analysed by ^{1}H NMR spectroscopy (Figure S7).

1.6 Catalyst Stability Tests

In the glovebox, a sample of the catalyst (40 µL of a 0.01M solution in THF) was transferred to an aluminium Tzero TGA crucible. The crucible was placed under vacuum, for 30 minutes, before being crimped in the glovebox with a hermetic seal. The crucible was then transferred to a TGA instrument and the following program run:

1. N_2 flow of 25.0 mL min^{-1}
2. Equilibrate at 30 °C
3. Heat to 140 °C
4. Isotherm at 140 °C, for 120 minutes
5. Cool to 30 °C

A small amount of mass loss was initially observed due to remaining solvent loss (~ 5 %). All catalysts retained ≥90 % mass after being held for 2 h at 140 °C.

1.7 PCHC Stability Test

A sample of PCHC was loaded into a crucible. The crucible was then transferred to a TGA instrument and the following program run:

1. N_2 flow of 25.0 mL min^{-1}
2. Equilibrate at 30 °C
3. Heat to 140 °C
4. Isotherm at 140 °C for 120 minutes
5. Cool to 30 °C

No mass loss of PCHC was observed.

1.8 Eyring Analysis for TGA Monitored Depolymerization

In the glovebox, PCHC (142 mg, 1.00 mmol) was added to a vial and dissolved in THF (1 mL). Mg(II)Co(II) was added from a stock solution (0.01 M in THF) to create a catalyst:polymer solution of $[PCHC]_0$:$[Cat]_0$ 2500:1. A

small sample of the catalyst:polymer solution (40 µL) was transferred to an aluminium Tzero TGA crucible. The crucible was placed under vacuum, for 30 minutes, before being crimped in the glovebox with a hermetic seal. The crucible was then transferred to a TGA instrument and depolymerization monitored at different, fixed temperatures (e.g. 110, 120, 125, 130 or 140 °C). The rate constant, k_{obs}, was extracted by taking the gradient of the linear fit of ln(mass/$mass_0$) *vs* time, at 50 % PCHC mass loss. Using the rate law (first order with respect to [catalyst] and [PCHC]) the value for k_d was determined.

The Eyring equation:

$$k_d = \frac{k_B T}{h} e^{\frac{-\Delta H^{\ddagger}}{RT}} e^{\frac{\Delta S^{\ddagger}}{R}}$$

Rearranged to give:

$$ln\frac{k_d}{T} = \frac{-\Delta H^{\ddagger}}{RT} + ln\frac{k_B}{h} + \frac{\Delta S^{\ddagger}}{R}$$

Therefore, Plots of $\frac{1}{T}$ vs. $ln\frac{k_d}{T}$:

$$gradient = \frac{-\Delta H^{\ddagger}}{R}$$

$$intercept = \frac{\Delta S^{\ddagger}}{R} + ln\frac{k_B}{h}$$

Values for the Mg(II)Co(II) catalyst:

$\Delta H^{\ddagger}$ = +136.8 ± 0.70 KJ mol^{-1}

$\Delta S^{\ddagger}$ = +97.6 ± 1.7 J mol^{-1}

$\Delta G^{\ddagger}_{140}$ = +96.5 ± 1.4 KJ mol^{-1}

1.9 Characterization of Mg(II)Fe(II) Catalyst Post Heating and Depolymerization

Under an N_2 atmosphere, PCHC (1.25 g, 8.80 mmol, 300 equiv.), dissolved in THF (5 mL), was added to an ampoule containing the Mg(II)Fe(II) catalyst (22 mg, 0.03 mmol, 1.00 equiv.). The reaction vessel was placed under dynamic vacuum (~10^{-2} mbar) for 1 h, to remove the solvent. After 1 h, it was placed under static vacuum and the reaction vessel heated to 140 °C for 2 h, after which atmospheric pressure was re-established using N_2. The Mg(II)Fe(II) catalyst was then characterized by cyclic voltammetry, MALDI-ToF mass spectrometry and IR spectroscopy (Figures S11-S13).

2. PCHC Characterization

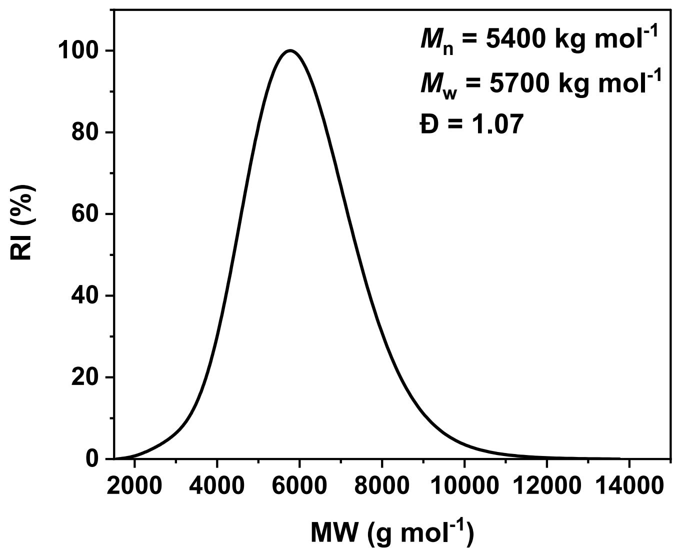

Figure S2. SEC data for the PCHC.

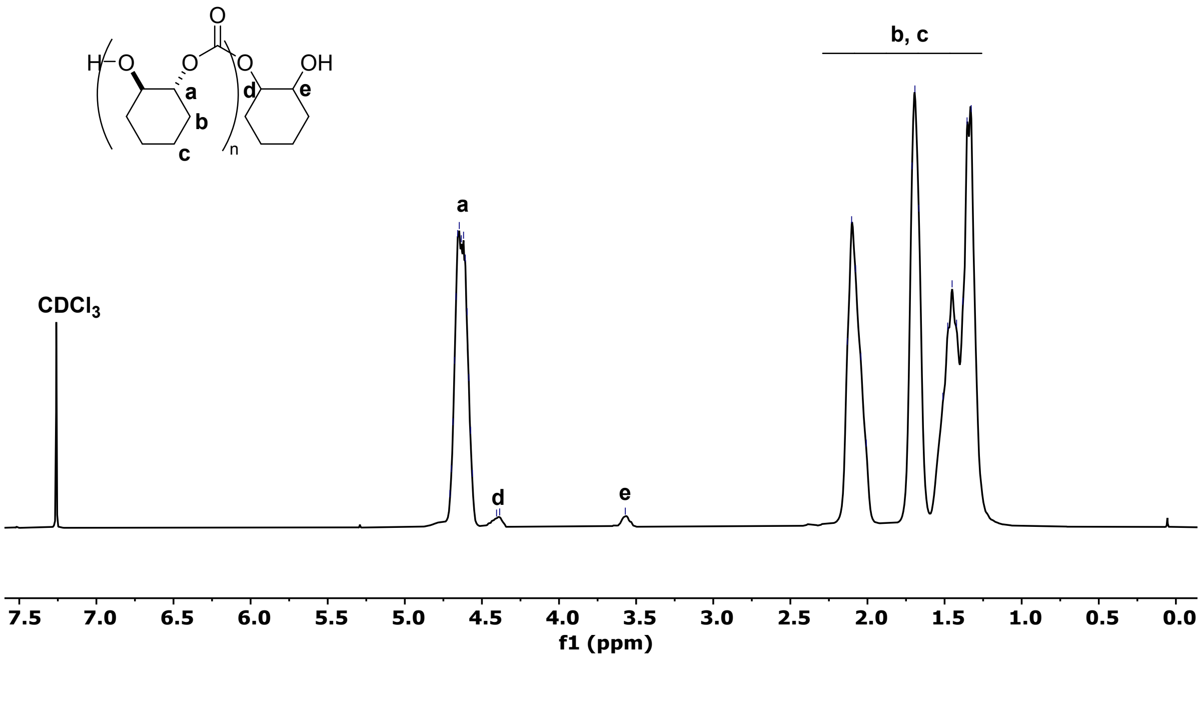

Figure S3. ^{1}H NMR Spectrum ($CDCl_3$) for the PCHC used in all depolymerizations.

3. Depolymerization Catalysis Data

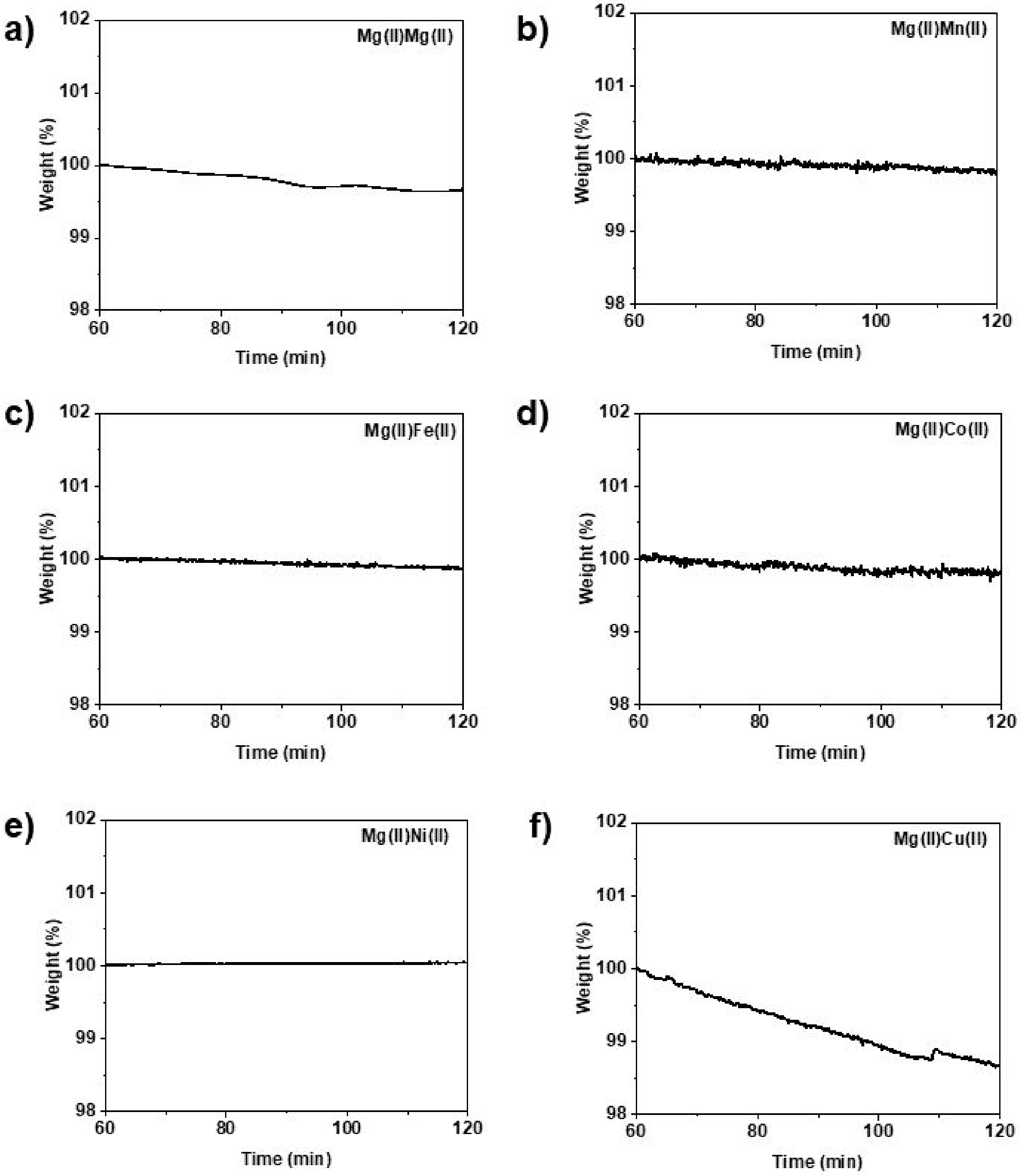

g)

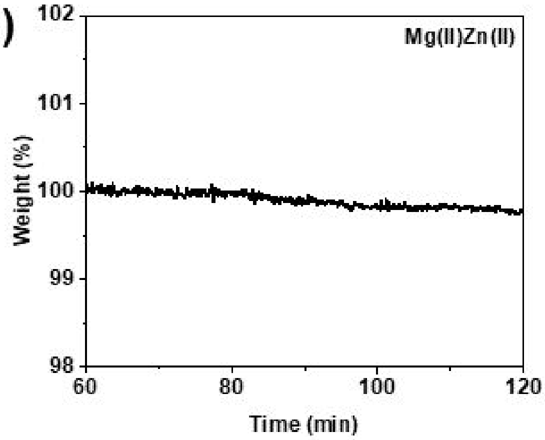

Figure S4. Mass loss vs time plots for catalysts demonstrating their stability (conditions: 140 °C for 2 h, under N_2). a) Mg(II)Mg(II) b) Mg(II)Mn(II)) c) Mg(II)Fe(II) d) Mg(II)Co(II) e) Mg(II)Ni(II) f) Mg(II)Cu(II) g) Mg(II)Zn(II).

Tests were run for 2 h, with data shown from 60 – 120 min. In the first few minutes of each run, ~ 5 % residual solvent was observed, thereafter no further mass loss occurred over 2 h, indicating the excellent stability of all catalysts.

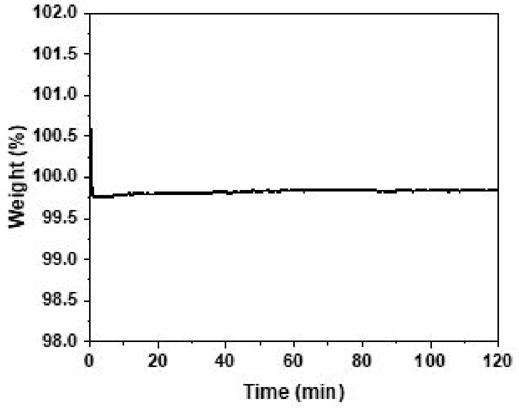

Figure S5. Mass loss vs time plots for PCHC with no catalyst present (Conditions: 140 °C, for 2 h, under N_2).

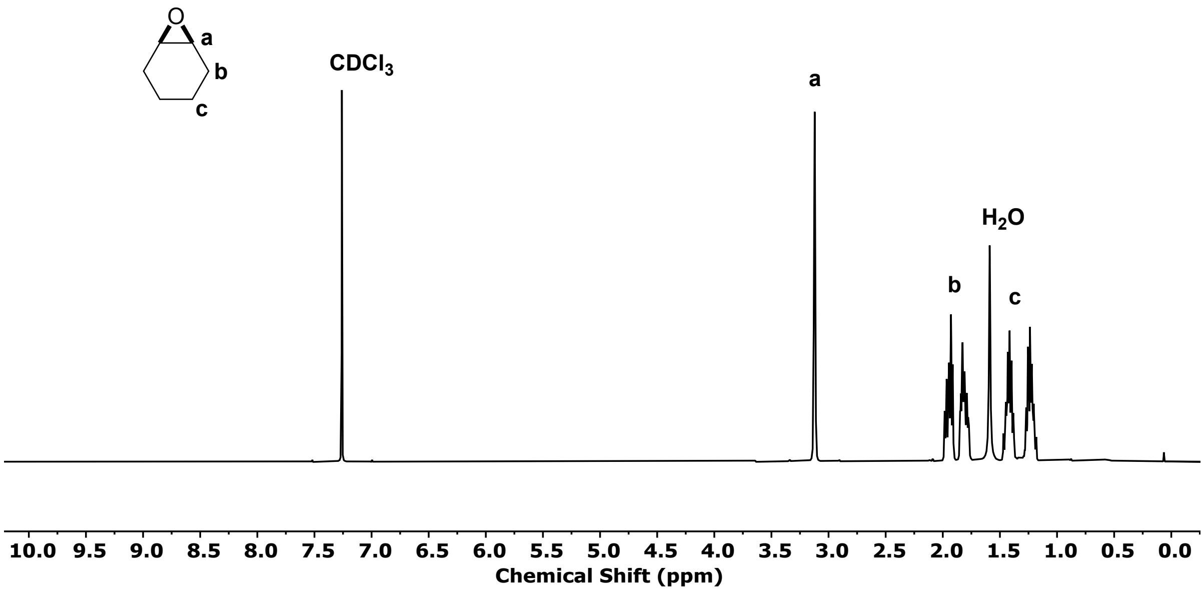

Figure S6. ^{1}H NMR Spectrum ($CDCl_3$) of CHO, isolated from the depolymerization of PCHC.

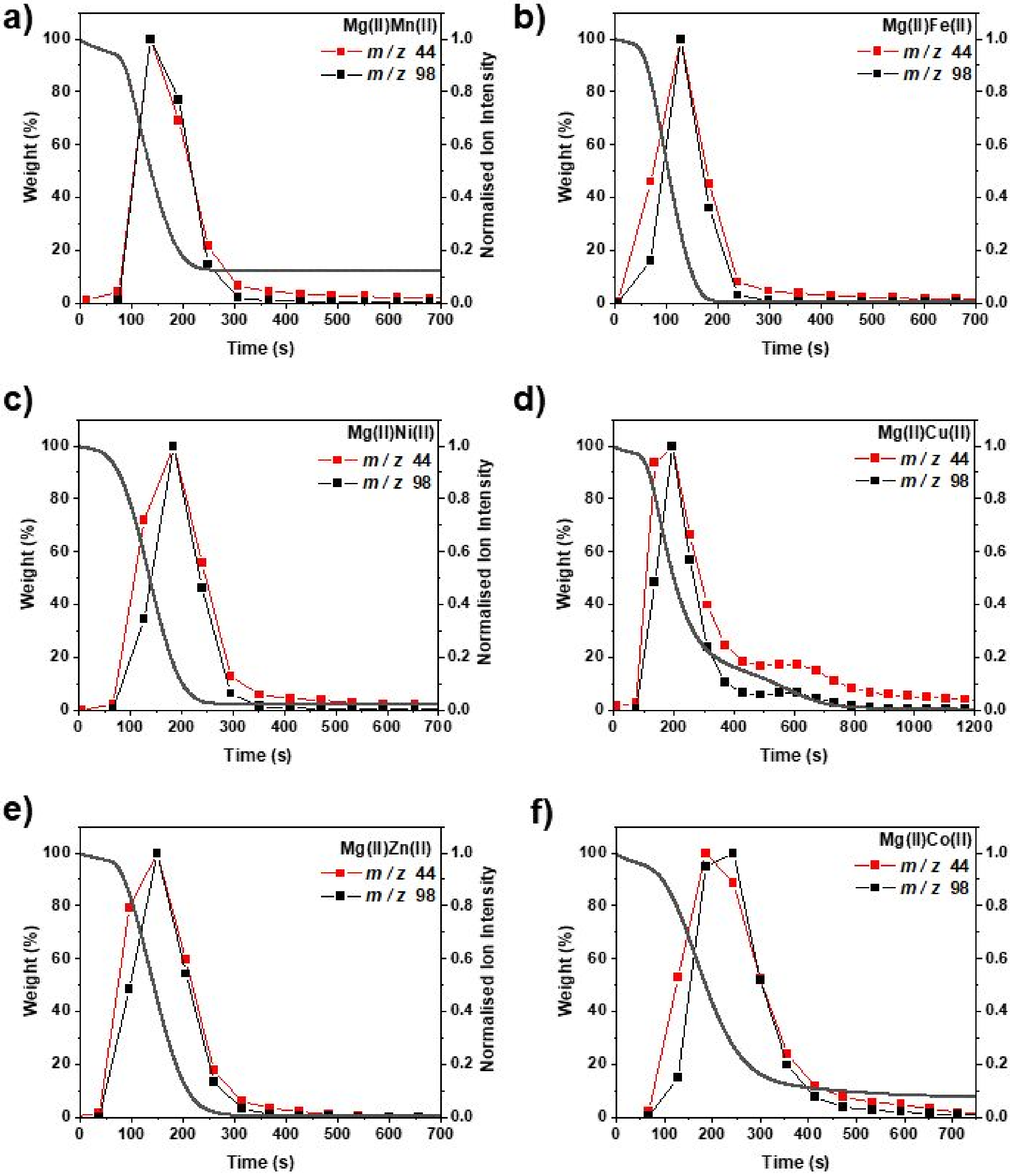

Figure S7. Mass loss and normalized ion intensity vs time for the depolymerization of PCHC. a) Mg(II)Mn(II) b) Mg(II)Fe(II) c) Mg(II)Ni(II) d) Mg(II)Cu(II) e) Mg(II)Zn(II). In all these experiments $[Mg(II)M(II)]_0$:$[PCHC]_0$ = 1:300. f) Depolymerization of PCHC by Mg(II)Co(II) where $[Mg(II)M(II)]_0$:$[PCHC]_0$ = 1:2500. The ions with *m/z* = 98 (black line) and 44 (red line) are assigned as CHO^+ (**M_r** = 98.13 g mol^{-1}) and CO_2^+ (**M_r** = 44 g mol^{-1}), respectively. TGA-MS data for Mg(II)Co(II) at $[Mg(II)M(II)]_0$:$[PCHC]_0$ = 1:300 has been previously published.[2]

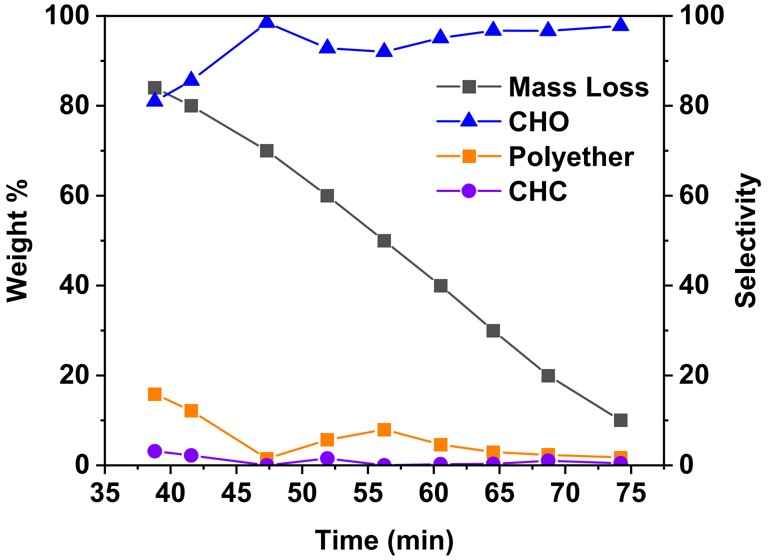

Figure S8. Identification of product distribution by isolation against % weight of PCHC. PCHC depolymerization performed at 140 °C, under N_2, using the Mg(II)Zn(II) catalyst. Mg(II)Zn(II):PCHC 1:300, mixed with pestle and mortar. ^{1}H NMR spectroscoy was conducted on samples removed at 10, 20, 30, 40, 50, 60, 70, 80 and 90 % mass loss.

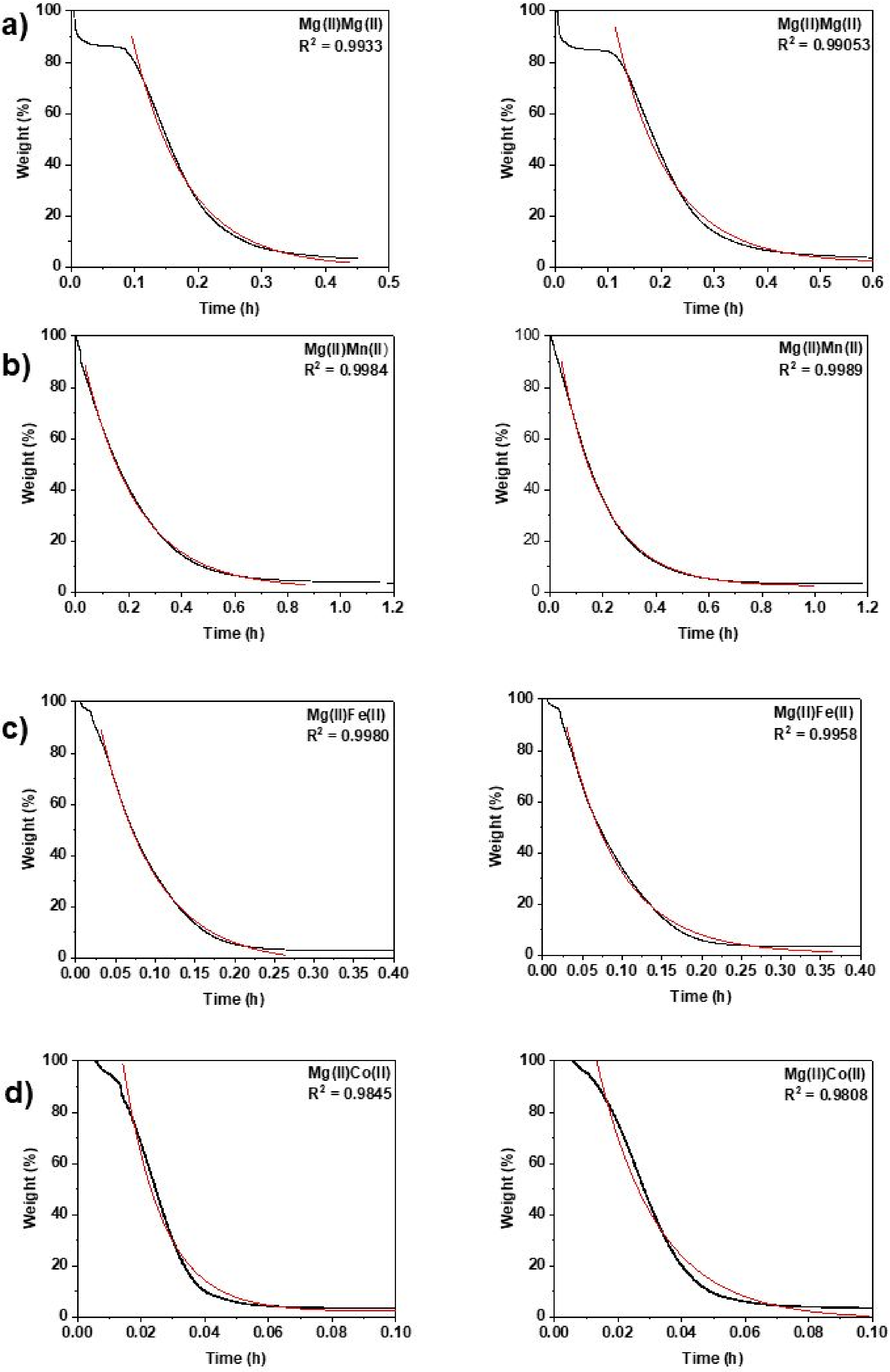
a)
b)
c)
d)
Mg(II)Mg(II)
R2 = 0.9933
R2 = 0.99053
Mg(II)Mn(II)
R2 = 0.9984
R2 = 0.9989
Mg(II)Fe(II)
R2 = 0.9980
R2 = 0.9958
Mg(II)Co(II)
R2 = 0.9845
R2 = 0.9808
Weight (%)
Time (h)

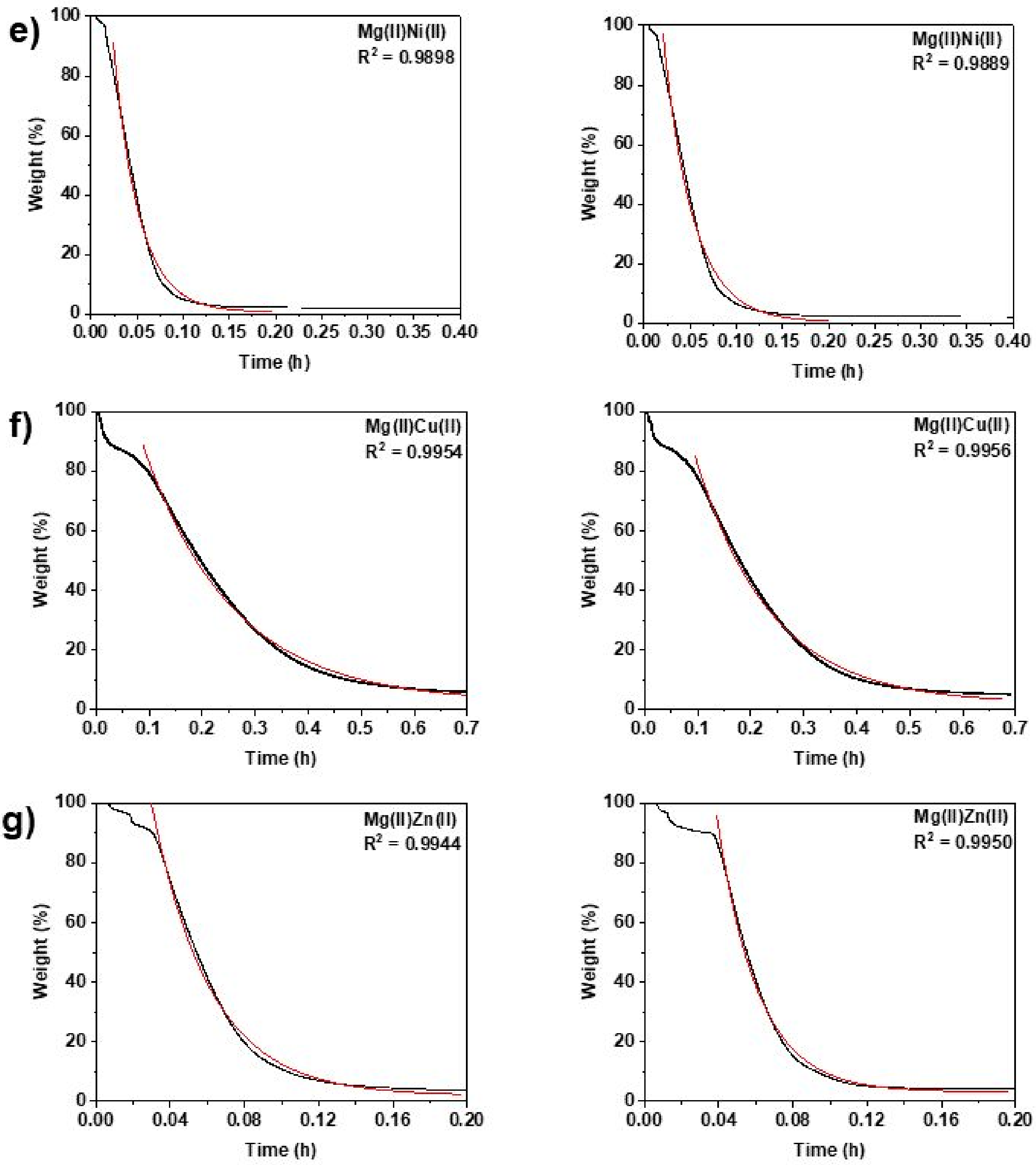

Figure S9. Mass loss vs time data for the depolymerization of PCHC fitted to a first order exponential decay. Experiments were repeated in triplicate, with data shown for two of the repeats. a) Mg(II)Mg(II) b) Mg(II)Mn(II) c) Mg(II)Fe(II) d) Mg(II)Co(II) e) Mg(II)Ni(II) f) Mg(II)Cu(II) g) Mg(II)Zn(II). Depolymerizations performed at 140 °C under N_2, $[cat]_0$:$[PCHC]_0$ 1:300.

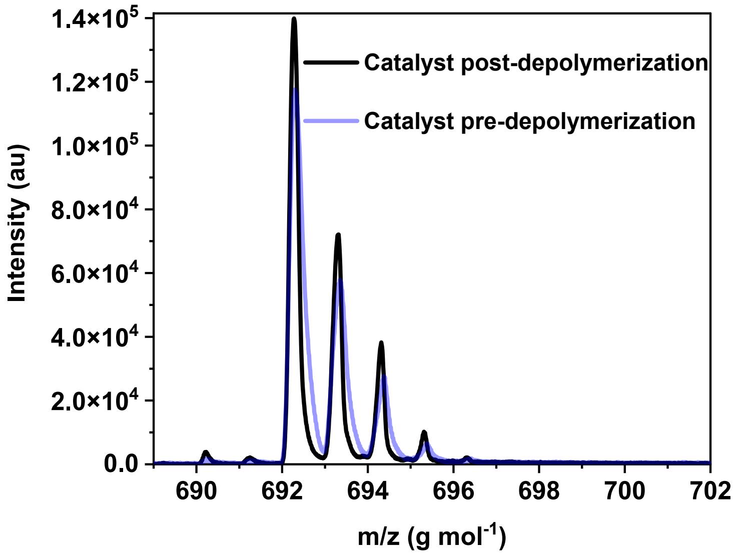

Figure S10. MALDI-TOF spectrum of catalyst before (purple) and after (black) depolymerization. Ions were detected as $[Mg(II)Co(II)L(OAc)]^{+}$ species in positive reflector mode. Data are reproduced from a prior report.[2]

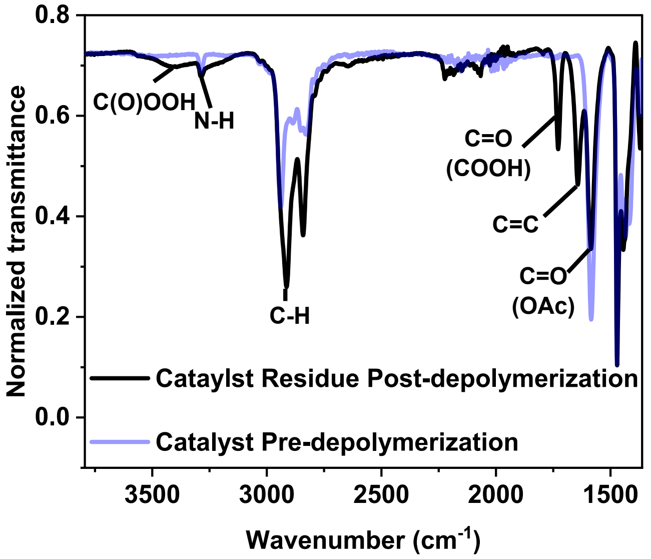

Figure S11. IR spectra of Mg(II)Co(II) before (purple) and after (black) depolymerization. The spectra show that the NH (3290 cm^{-1}), CH (2841 cm^{-1}), and C=O (OAc, 1531cm^{-1}) stretches are unchanged post depolymerization, albeit with the complex exhibiting a less intense acetate stretch. Post depolymerization, new stretches appear at 3400 cm^{-1} (broad) 1728 cm^{-1} and 1640 cm^{-1}. These stretches do not correspond to residual polymer (1750 cm^{-1}), *trans*-CHC (1820 cm^{-1}) or *cis* CHC (1804 cm^{-1}) The stretches at 3400 cm^{-1} and 1728 cm^{-1} are, thus, attributed to a carboxylic acid species, whilst the stretch at 1640 cm^{-1} remains unassigned. Data reproduced from previous work.[2]

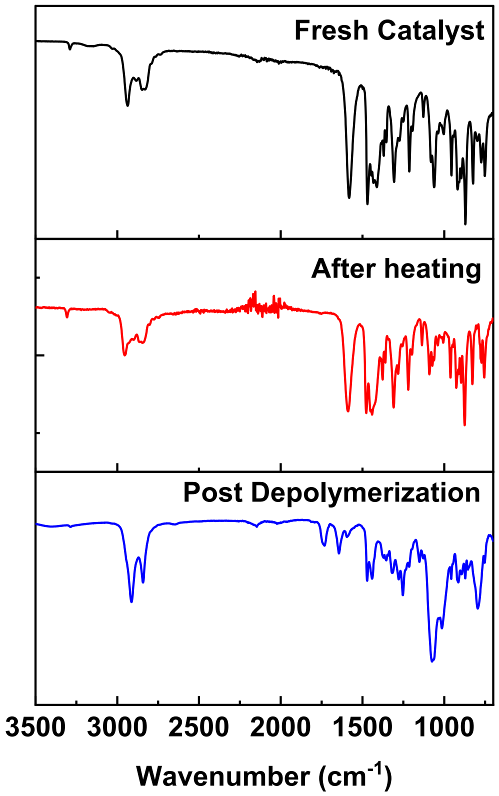

Figure S12. IR Spectra of the Mg(II)Fe(II) catalyst characterized after heating at 140 °C for 2 h and post depolymerization reaction**.** Depolymerization performed at 140 °C under N_2, $[cat]_0$:$[PCHC]_0$ 1:300. New peaks in the post depolymerization sample suggest some catalyst decomposition (different to the Mg(II)Co(II) catalyst).

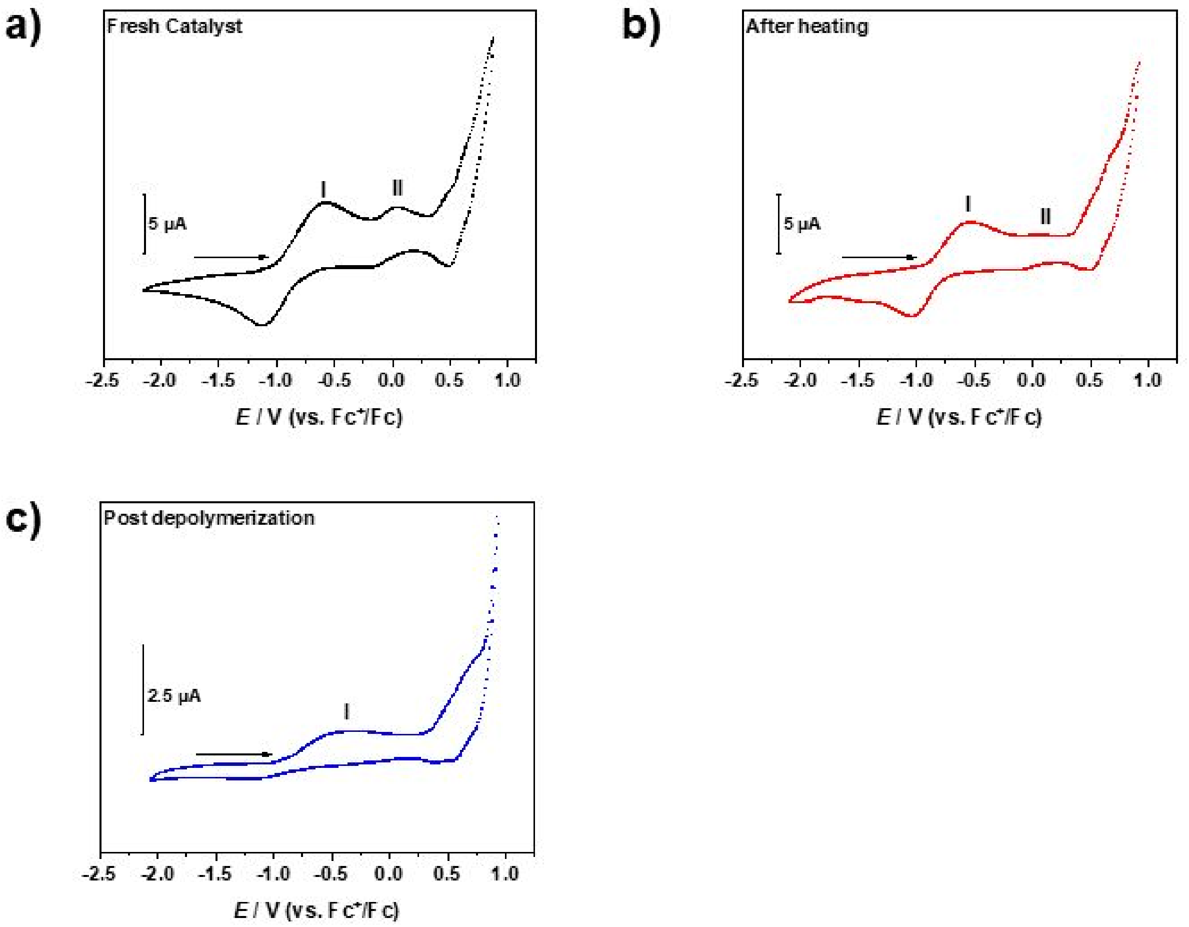

Figure S13. Cyclic voltammograms for the Mg(II)Fe(II) catalyst. a) fresh catalyst, b) after heating at 140 °C for 2 h and c) post depolymerization reaction**.** Depolymerization performed at 140 °C under N_2, $[cat]_0$:$[PCHC]_0$ 1:300. The post depolymerization sample has clearly changed suggested Fe-centred redox processes occur upon heating/reactions.

a) $E^{I}_{1/2}$ = -0.864 V
   $E^{II}_{1/2}$ = -0.050 V
b) $E^{I}_{1/2}$ = -0.799 V
   $E^{II}_{1/2}$ = 0.055 V
c) $E^{I}_{1/2}$ = -0.784 V

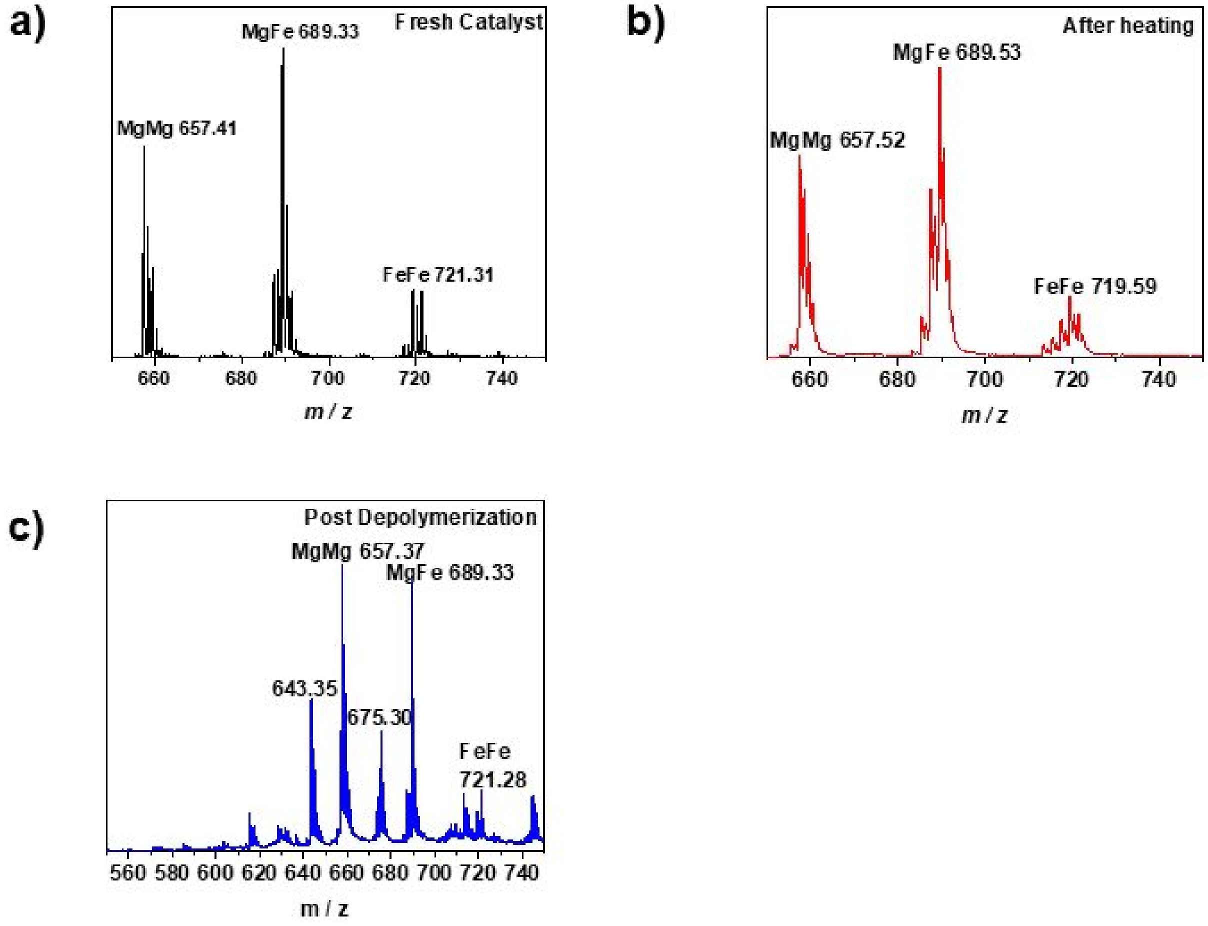

Figure S14. MALDI-ToF spectra for the Mg(II)Fe(II) catalyst. a) Fresh catalyst, b) Catalyst after heating at 140 °C for 2 h and c) Catalyst post depolymerization reaction. Depolymerization performed at 140 °C under N_2, $[cat]_0$:$[PCHC]_0$ 1:300. Additional unassigned peaks are observed in the spectrum of the catalyst post depolymerization suggesting some catalyst decomposition (different to the results with the Mg(II)Co(II) catalyst).

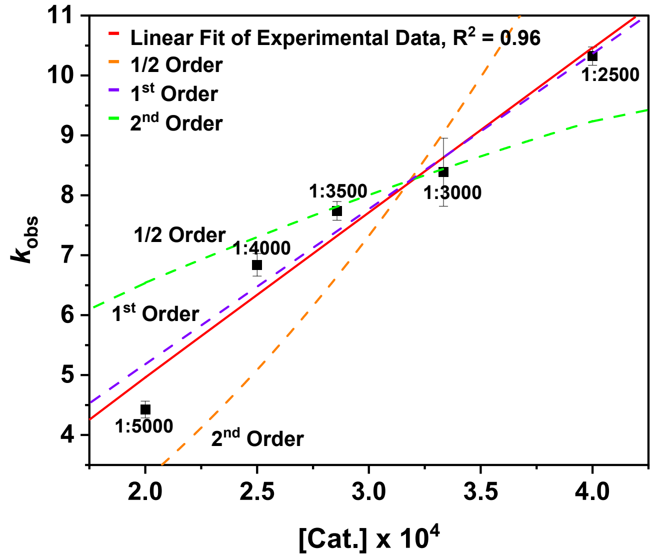

Figure S15. Plot of k_{obs} vs. [cat]. The ratio $[cat]_0$:$[PCHC]_0$ is indicated for each data point. PCHC depolymerization performed at 140 °C, under N_2. Dashed lines on the plot show the fit for a ½ order (green), 1st order (purple) and 2nd order (orange) relationship, along with the best fit line of the experimental data (red continuous line). The experimental data fits most closely to a linear line of best fit, indicating a first order dependence of the rate on catalyst concentration.

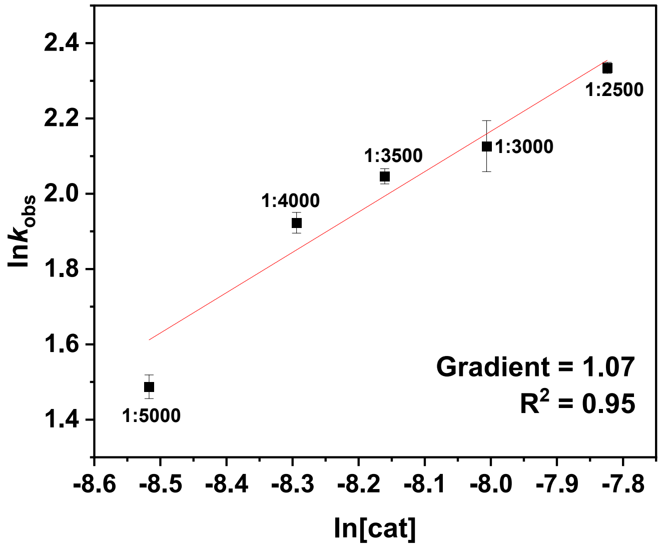

Figure S16. Determination of the order in catalyst concentration. Plot of lnk_{obs} vs ln[cat] and a linear fit. The ratio $[cat]_0$:$[PCHC]_0$ is indicated for each data point. PCHC depolymerization performed at 140 °C, under N_2.

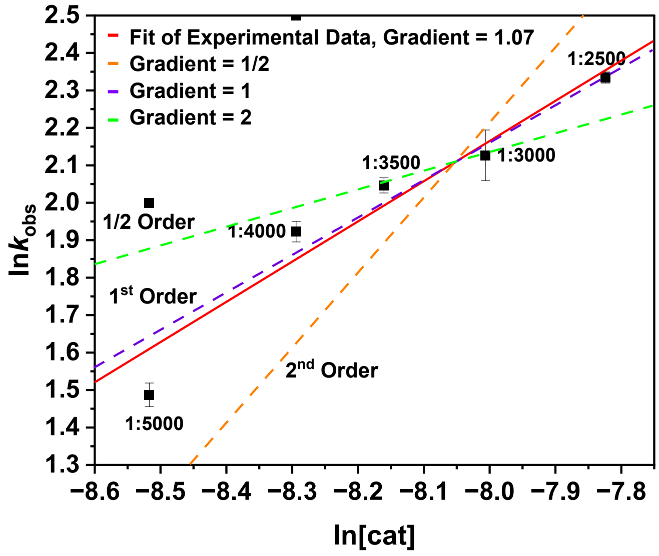

Figure S17. Determination of the order in catalyst concentration. Plot of lnk_{obs} vs ln[cat]. The ratio $[cat]_0$:$[PCHC]_0$ is indicated for each data point. PCHC depolymerization performed at 140 °C, under N_2. Dashed lines on the plot show the fit for a ½ order (green), 1st order (purple) and 2nd order (orange) relationship, along with a linear fit of the experimental data (red continuous line). The experimental data fits most closely to a gradient of 1, indicating a first order dependence of the rate on catalyst concentration.

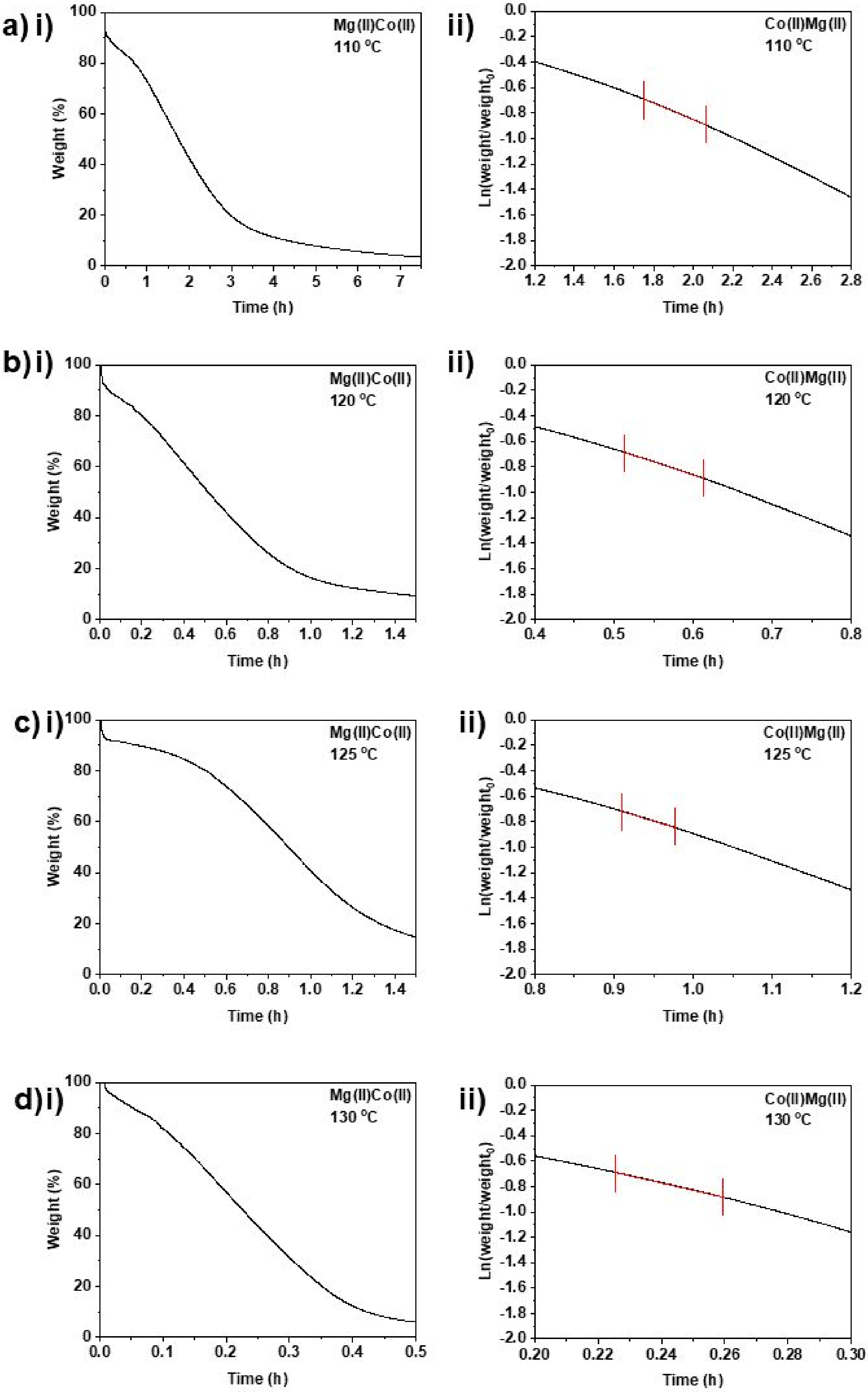
a) i)
Mg(II)Co(II)
110 °C
Weight (%)
Time (h)
ii)
Co(II)Mg(II)
110 °C
Ln(weight/weight$_0$)
Time (h)
b) i)
Mg(II)Co(II)
120 °C
Weight (%)
Time (h)
ii)
Co(II)Mg(II)
120 °C
Ln(weight/weight$_0$)
Time (h)
c) i)
Mg(II)Co(II)
125 °C
Weight (%)
Time (h)
ii)
Co(II)Mg(II)
125 °C
Ln(weight/weight$_0$)
Time (h)
d) i)
Mg(II)Co(II)
130 °C
Weight (%)
Time (h)
ii)
Co(II)Mg(II)
130 °C
Ln(weight/weight$_0$)
Time (h)

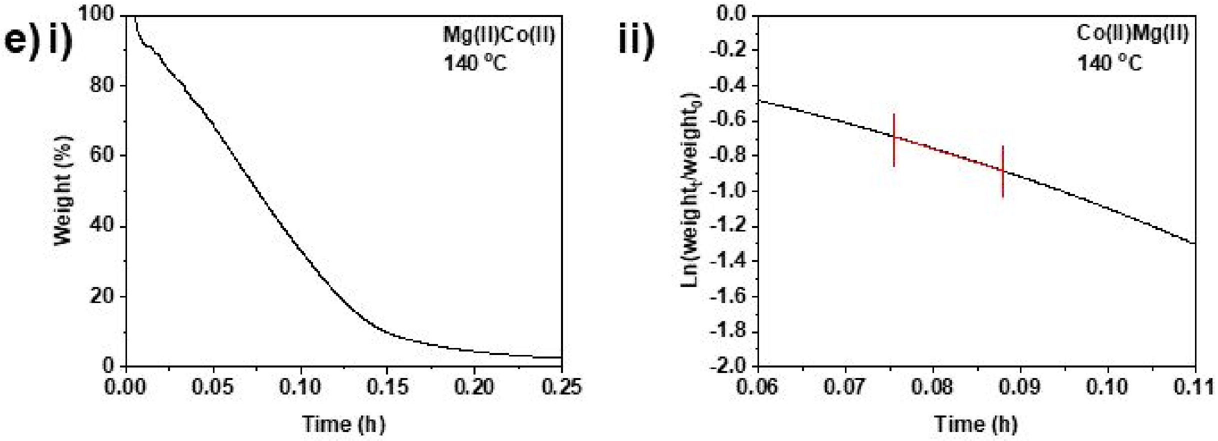

Figure S18. Depolymerizations of PCHC using the Mg(II)Co(II) catalyst and data used for Eyring analysis. Depolymerization performed under N_2, $[cat]_0$:$[PCHC]_0$ 1:2500, at different temperatures. a) 110 °C, b) 120 °C, c) 125 °C, d) 130 °C, e) 140 °C. i) Plots of mass loss vs time for PCHC depolymerization. ii) Selected regions of ln(weight/weight$_0$) vs time plots with linear fits, where k_{obs} is reported at 50 % PCHC mass (k_{obs} = gradient). All mass loss data was repeated in triplicate and used to determine errors in k_{obs} values.

Table S1. Literature values for the hydrolysis constants of the M(II) cations.[5]

Metal Cation	Hydrolysis Constant
Mg(II)	10.59
Mn(II)	9.5
Fe(II)	9.65
Co(II)	9.86
Cu(II)	7.53
Zn(II)	8.96

$pK_h = -\log K_{xy}$

$x\,M^{z+} + y\,H_2O \leftrightarrow M_x(OH)_y^{(xz-y)+} + y\,H^+$

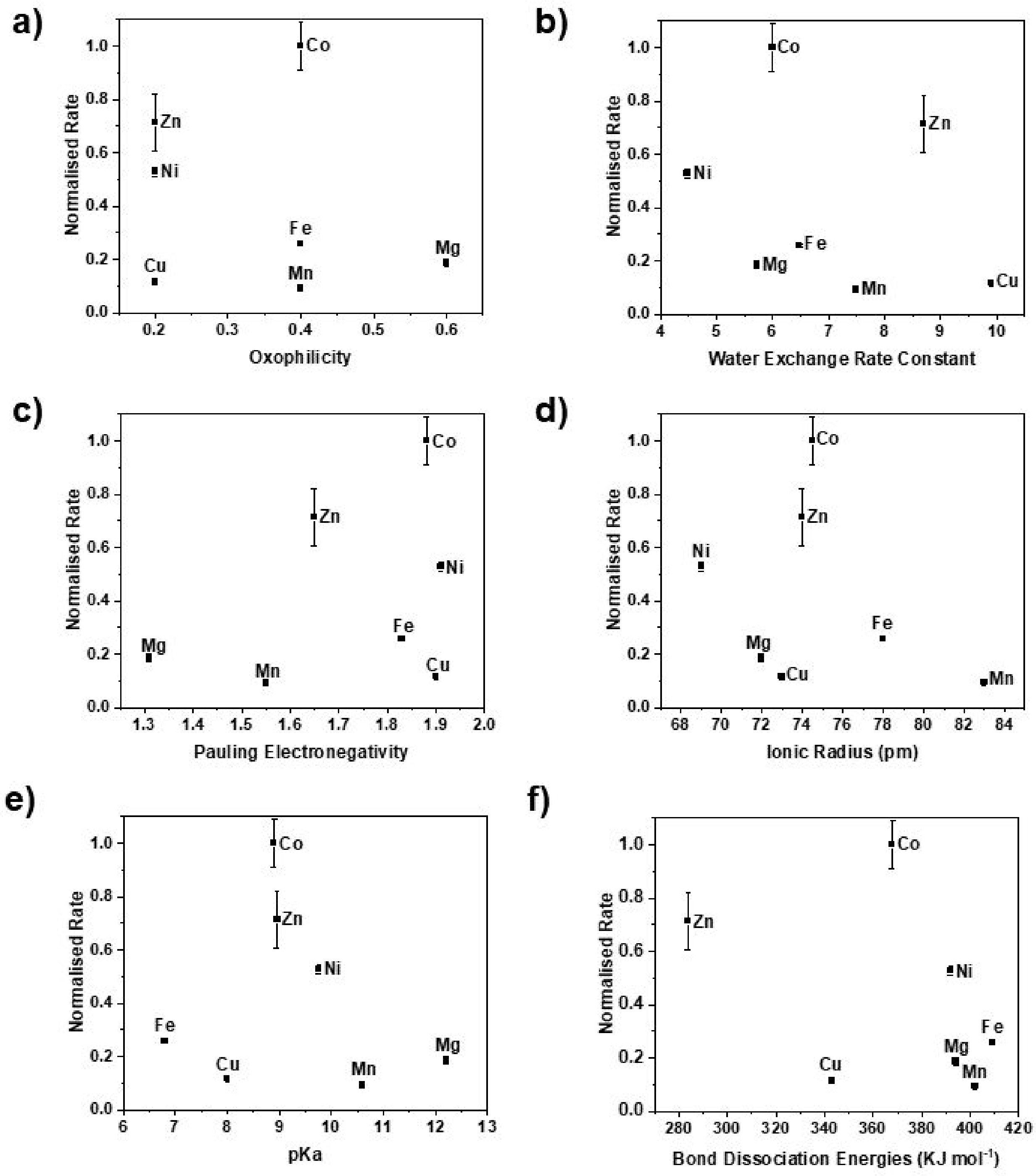

Figure S19. Plots of the normalised k_{obs} for depolymerization against various proxies for metal alkoxide nucleophilicity: a) Oxophilicity[6] b) Water exchange rate constant[5] c) Pauling electronegativity[7] d) Ionic radius[8] e) pKa of the transition metal aqua complex[9] and f) Bond dissociation energies[10].

4. References

(1) Deacy, A. C.; Moreby, E.; Phanopoulos, A.; Williams, C. K. Co(III)/Alkali-Metal(I) Heterodinuclear Catalysts for the Ring-Opening Copolymerization of CO_2 and Propylene Oxide. *J. Am. Chem. Soc.* **2020**, *142* (45), 19150-19160.

(2) McGuire, T. M.; Deacy, A. C.; Buchard, A.; Williams, C. K. Solid-State Chemical Recycling of Polycarbonates to Epoxides and Carbon Dioxide Using a Heterodinuclear Mg(II)Co(II) Catalyst. *J. Am. Chem. Soc.* **2022**, *144* (40), 18444-18449.

(3) Kember, M. R.; Knight, P. D.; Reung, P. T. R.; Williams, C. K. Highly Active Dizinc Catalyst for the Copolymerization of Carbon Dioxide and Cyclohexene Oxide at One Atmosphere Pressure. *Angew. Chem. Int. Ed.* **2009**, *48* (5), 931-933.

(4) Reis, N. V.; Deacy, A. C.; Rosetto, G.; Durr, C. B.; Williams, C. K. Heterodinuclear Mg (II) M (II)(M= Cr, Mn, Fe, Co, Ni, Cu and Zn) Complexes for the Ring Opening Copolymerization of Carbon Dioxide/Epoxide and Anhydride/Epoxide. *Chem. Eur. J.* **2022**, *28* (14), e202104198.

(5) Kobayashi, S.; Nagayama, S.; Busujima, T. Lewis Acid Catalysts Stable in Water. Correlation between Catalytic Activity in Water and Hydrolysis Constants and Exchange Rate Constants for Substitution of Inner-Sphere Water Ligands. *J. Am. Chem. Soc.* **1998**, *120* (32), 8287-8288.

(6) Kepp, K. P. A Quantitative Scale of Oxophilicity and Thiophilicity. *Inorg. Chem.* **2016**, *55* (18), 9461-9470.

(7) Emsley, J., Clarendon Press, "The Elements." **1998**, ISBN: 0198558198, The Elements by John Emsley

(8) Shannon, R. D. Revised effective ionic radii and systematic studies of interatomic distances in halides and chalcogenides. *Acta Cryst. Section A* **1976**, *32* (5), 751-767.

(9) Perrin, D. D., Elsevier, 2nd Edition, "Ionisation Constants of Inorganic Acids and Bases in Aqueous Solution." **1982**, ISBN: 9781483284613.

(10) Luo, Y.-R., Taylor Francis, CRC, Boca Raton. "Comprehensive Handbook of Chemical Bond Energies." **2007**, https://doi.org/10.1201/9781420007282.
